# Surveillance for Violent Deaths — National Violent Death Reporting System, 32 States, 2016

**DOI:** 10.15585/mmwr.ss.6809a1

**Published:** 2019-10-04

**Authors:** Allison Ertl, Kameron J. Sheats, Emiko Petrosky, Carter J. Betz, Keming Yuan, Katherine A. Fowler

**Affiliations:** 1 Division of Violence Prevention, National Center for Injury Prevention and Control, CDC

## Abstract

**Problem/Condition:**

In 2016, approximately 65,000 persons died in the United States as a result of violence-related injuries. This report summarizes data from CDC’s National Violent Death Reporting System (NVDRS) regarding violent deaths from 32 U.S. states for 2016. Results are reported by sex, age group, race/ethnicity, type of location where injured, method of injury, circumstances of injury, and other selected characteristics.

**Period Covered:**

2016.

**Description of System:**

NVDRS collects data regarding violent deaths obtained from death certificates, coroner/medical examiner reports, law enforcement reports, and secondary sources (e.g., child fatality review team data, Supplementary Homicide Reports, hospital data, and crime laboratory data). This report includes data collected from 32 states for 2016 (Alaska, Arizona, Colorado, Connecticut, Georgia, Hawaii, Illinois, Indiana, Iowa, Kansas, Kentucky, Maine, Maryland, Massachusetts, Michigan, Minnesota, New Hampshire, New Jersey, New Mexico, New York, North Carolina, Ohio, Oklahoma, Oregon, Pennsylvania, Rhode Island, South Carolina, Utah, Vermont, Virginia, Washington, and Wisconsin). NVDRS collates information for each death and links deaths that are related (e.g., multiple homicides, homicide followed by suicide, or multiple suicides) into a single incident.

**Results:**

For 2016, NVDRS captured 40,374 fatal incidents involving 41,466 deaths in the 32 states included in this report. The majority (62.3%) of deaths were suicides, followed by homicides (24.9%), deaths of undetermined intent (10.8%), legal intervention deaths (1.2%) (i.e., deaths caused by law enforcement and other persons with legal authority to use deadly force acting in the line of duty, excluding legal executions), and unintentional firearm deaths (<1.0%). (The term legal intervention is a classification incorporated into the *International Classification of Diseases, Tenth Revision* [*ICD-10*] and does not denote the lawfulness or legality of the circumstances surrounding a death caused by law enforcement.) Demographic patterns varied by manner of death. Suicide rates were highest among males, non-Hispanic American Indians/Alaska Natives, non-Hispanic whites, adults aged 45–64 years, and men aged ≥75 years. The most common method of injury was a firearm among males and poisoning among females. Suicides were most often preceded by a mental health, intimate partner, substance abuse, or physical health problem or a recent or impending crisis during the previous or upcoming 2 weeks. Homicide rates were highest among males and persons aged <1 year and 15–44 years. Among males, non-Hispanic blacks accounted for most homicides and had the highest rate of any racial/ethnic group. The most common method of injury was a firearm. Homicides were most often precipitated by an argument or conflict, occurred in conjunction with another crime, or for females, were related to intimate partner violence. When the relationship between a homicide victim and a suspected perpetrator was known, the suspect was most frequently an acquaintance/friend among males and a current or former intimate partner among females. Legal intervention death rates were highest among men aged 20–44 years, and the rate among non-Hispanic black males was three times the rate among non-Hispanic white males. Precipitating circumstances for legal intervention deaths most frequently were an alleged criminal activity in progress, reported use of a weapon by the victim in the incident, a mental health or substance abuse problem (other than alcohol abuse), an argument or conflict, or a recent or impending crisis. Unintentional firearm deaths were more frequent among males, non-Hispanic whites, and persons aged 15–24 years. These deaths most often occurred while the shooter was playing with a firearm and most often were precipitated by a person unintentionally pulling the trigger or mistakenly thinking the firearm was unloaded. Rates of deaths of undetermined intent were highest among males, particularly non-Hispanic black and American Indian/Alaska Native males, and adults aged 25–64 years. Substance abuse, mental health problems, physical health problems, and a recent or impending crisis were the most common circumstances preceding deaths of undetermined intent. In 2016, a total of 3,655 youths aged 10–24 years died by suicide. The majority of these decedents were male, non-Hispanic white, and aged 18–24 years. Most decedents aged 10–17 years died by hanging/strangulation/suffocation (49.3%), followed by a firearm (40.4%), and suicides among this age group were most often precipitated by mental health, family relationship, and school problems. Most suicides among decedents aged 18–24 years were by a firearm (46.2%), followed by hanging/strangulation/suffocation (37.4%), and were precipitated by mental health, substance abuse, intimate partner, and family problems. A recent crisis, an argument or conflict, or both were common precipitating circumstances among all youth suicide decedents.

**Interpretation:**

This report provides a detailed summary of data from NVDRS for 2016. Suicides rates were highest among non-Hispanic American Indian/Alaska Native and white males, whereas homicide rates were highest among non-Hispanic black males. Mental health problems, intimate partner problems, interpersonal conflicts, and acute life stressors were primary precipitating events for multiple types of violent deaths, including suicides among youths aged 10–24 years.

**Public Health Action:**

NVDRS data are used to monitor the occurrence of violence-related fatal injuries and assist public health authorities in the development, implementation, and evaluation of programs and policies to reduce and prevent violent deaths. For example, Utah VDRS data were used to help identify suicide risk factors among youths aged 10–17 years, Rhode Island VDRS suicide data were analyzed to identify precipitating circumstances of youth suicides over a 10-year period, and Kansas VDRS data were used by the Kansas Youth Suicide Prevention Task Force. In 2019, NVDRS expanded data collection to include all 50 states, Puerto Rico, and the District of Columbia. This expansion is essential to public health efforts to reduce violent deaths.

## Introduction

In 2016, violence-related injuries led to approximately 65,000 deaths in the United States (*1*). Suicide was the 10th leading cause of death overall in the United States and disproportionately affected young and middle-aged populations. Suicide was among the top two leading causes of death for persons aged 10–34 years and among the top four for persons aged 35–54 years. Non-Hispanic American Indian/Alaska Native and non-Hispanic white males were disproportionately affected by suicide.

Homicide was the 16th leading cause of death overall in the United States but disproportionately affected young persons (*1*). Homicide was the fourth leading cause of death for persons aged 1–14 years and the third leading cause of death for persons aged 15–34 years. Young non-Hispanic black males were disproportionately affected by homicide, which was the leading cause of death for non-Hispanic black males aged 15–34 years and the second leading cause of death for non-Hispanic black males aged 1–4 and 10–14 years.

Public health authorities require accurate, timely, and complete surveillance data to better understand and ultimately prevent the occurrence of violent deaths in the United States (*2*). In 2000, in response to an Institute of Medicine* report noting the need for a national fatal intentional injury surveillance system (*3*), CDC began planning to implement the National Violent Death Reporting System (NVDRS) (*2*). The goals of NVDRS are to

- collect and analyze timely, high-quality data for monitoring the magnitude and characteristics of violent deaths at national, state, and local levels;
- ensure data are disseminated routinely and expeditiously to public health officials, law enforcement officials, policymakers, and the public;
- ensure data are used to develop, implement, and evaluate programs and strategies that are intended to reduce and prevent violent deaths and injuries at national, state, and local levels; and
- build and strengthen partnerships among organizations and communities at national, state, and local levels to ensure that data are collected and used to reduce and prevent violent deaths and injuries.

NVDRS is a state-based active surveillance system that collects data on the characteristics and circumstances associated with violence-related deaths in participating states and territories. Deaths include homicides, suicides, legal intervention deaths (i.e., deaths caused by law enforcement acting in the line of duty and other persons with legal authority to use deadly force, excluding legal executions), unintentional firearm deaths, and deaths of undetermined intent that might have been due to violence.^†^ (The term legal intervention is a classification incorporated into the *International Classification of Diseases, Tenth Revision* [*ICD-10*] and does not denote the lawfulness or legality of the circumstances surrounding a death caused by law enforcement.) NVDRS data are used to assist development, implementation, and evaluation of programs and strategies designed to reduce and prevent violent deaths at national, state, and local levels.

Before implementation of NVDRS, single data sources (e.g., death certificates) provided only limited information and few circumstances from which to understand patterns of violent deaths. NVDRS fills this surveillance gap by providing more detailed information. NVDRS is the first system to 1) provide detailed information on circumstances precipitating violent deaths, 2) link multiple source documents so that each incident can contribute to the study of patterns of violent deaths, and 3) link multiple deaths that are related (e.g., multiple homicides, suicide pacts, or homicide followed by suicide of the suspected perpetrator).

NVDRS data collection began in 2003 with six participating states (Maryland, Massachusetts, New Jersey, Oregon, South Carolina, and Virginia). Seven states (Alaska, Colorado, Georgia, North Carolina, Oklahoma, Rhode Island, and Wisconsin) began data collection in 2004, three (Kentucky, New Mexico, and Utah) in 2005, two (Ohio and Michigan) in 2010, and 14 (Arizona, Connecticut, Hawaii, Illinois, Indiana, Iowa, Kansas, Maine, Minnesota, New Hampshire, New York, Pennsylvania, Vermont, and Washington) in 2015. Eight states (Alabama, California, Delaware, Louisiana, Missouri, Nebraska, Nevada, and West Virginia), the District of Columbia, and Puerto Rico began data collection in 2017.^§^ In 2018, NVDRS received funding for nationwide expansion, and the remaining 10 states (Arkansas, Florida, Idaho, Mississippi, Montana, North Dakota, South Dakota, Tennessee, Texas, and Wyoming) began data collection in 2019 (Figure). CDC now provides funding for participation to all 50 states, Puerto Rico, and the District of Columbia.

**FIGURE:**
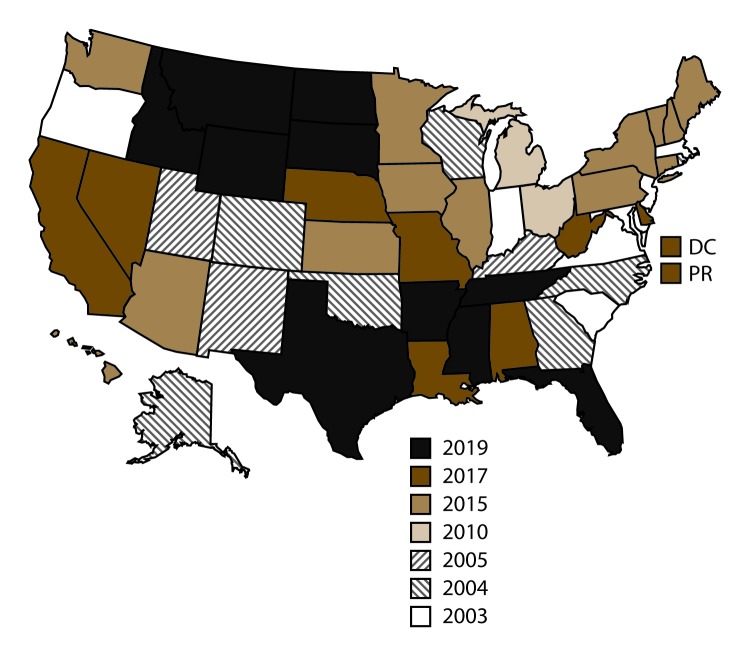
States participating in the National Violent Death Reporting System, by year of initial data collection* — United States and Puerto Rico, 2003–2109 **Abbreviations:** DC = District of Columbia; PR = Puerto Rico. * California began collecting data for a subset of violent deaths in 2005 but ended data collection in 2009. In 2017, California resumed data collection. Michigan collected data for a subset of violent deaths during 2010–2013 and collected statewide data beginning in 2014. In 2016, Illinois, Pennsylvania, and Washington began collecting data on violent deaths in a subset of counties that represents ≥80% of all violent deaths in the states or in counties where ≥1,800 violent deaths occur.

This report summarizes NVDRS data on violent deaths collected by 32 states in 2016 and highlights the finding that during this time suicide was the second leading cause of death among youths aged 10–24 years (*1*). During 1999–2016, the unadjusted suicide rate among youths aged 10–17 years increased approximately 48%, from 3.1 to 4.6 per 100,000 population (*1*). Among youths aged 18–24 years, the unadjusted suicide rate increased 27.1%, from 11.8 to 15.0 per 100,000 population, during this period (*1*). The increasing suicide rate among youths warrants more comprehensive understanding of the characteristics and circumstances of these deaths to develop suicide prevention for this population.

Twenty-nine of the participating 32 states collected information on all violent deaths occurring in their state (Alaska, Arizona, Colorado, Connecticut, Georgia, Hawaii, Indiana, Iowa, Kansas, Kentucky, Maine, Maryland, Massachusetts, Michigan, Minnesota, New Hampshire, New Jersey, New Mexico, New York, North Carolina, Ohio, Oklahoma, Oregon, Rhode Island, South Carolina, Utah, Vermont, Virginia, and Wisconsin). Three states (Illinois, Pennsylvania, and Washington) joined NVDRS with plans to collect data on violent deaths in a subset of counties that represents ≥80% of all violent deaths in the state or in counties where ≥1,800 violent deaths occur. In 2016, these states reported data on ≥80% of violent deaths in their state. Because <100% of violent deaths were targeted for data collection, data from these three states might not be fully representative of all violent deaths occurring in the state. However, these states were included in the national data set because they captured the majority of violent deaths in their states. In 2016, the 32 states accounted for 58% of the U.S. population (*1*,*4*). NVDRS data are updated annually and are available to the public through CDC’s Web-based Injury Statistics Query and Reporting System (WISQARS)^¶^ at https://www.cdc.gov/injury/wisqars/nvdrs.html. Case-level NVDRS data are available to applicants who meet eligibility requirements via access to the NVDRS Restricted Access Database (https://www.cdc.gov/ViolencePrevention/NVDRS/RAD.html).

## Methods

NVDRS compiles information from three required data sources: death certificates, coroner/medical examiner reports, and law enforcement reports. Certain participating states also collect information from secondary sources (e.g., child fatality review team data, Supplementary Homicide Reports, and crime laboratory data). NVDRS combines information for each death and links deaths that are related (e.g., multiple homicides, homicide followed by suicide, or multiple suicides) into a single incident. The ability to analyze linked data can provide more comprehensive understanding of violent deaths. This report presents selected data for 2016. Additional data from 2016 are available (Supplementary Tables, https://stacks.cdc.gov/view/cdc/78990).

In NVDRS, a violent death is defined as a death resulting from the intentional use of physical force or power, threatened or actual, against oneself, another person, or a group or community (*5*). Information also is collected about unintentional firearm deaths (i.e., a death resulting from a penetrating injury or gunshot wound from a weapon that uses a powder charge to fire a projectile when there was a preponderance of evidence that the shooting was not intentionally directed at the victim) and deaths of undetermined intent (i.e., a death that results from the use of force or power against oneself or another person for which the evidence indicating one manner of death is no more compelling than evidence indicating another). NVDRS cases are coded on the basis of *ICD-10* (*6*) or the manner of death assigned by the coroner/medical examiner or law enforcement. Cases are included if they are assigned *ICD-10* codes (Box 1) or a manner of death specified in at least one of the three primary data sources consistent with NVDRS case definitions.

BOX 1 ***International Classification of Diseases, Tenth Revision *(*ICD-10*) codes used in the National Violent Death Reporting System**

**Table:** 

Manner of death	Death ≤1 year after injury	Death >1 year after injury	Death any time after injury
Intentional self-harm (suicide)	X60–X84	Y87.0	U03 (attributable to terrorism)
Assault (homicide)	X85–X99, Y00–Y09	Y87.1	U01, U02 (attributable to terrorism)
Event of undetermined intent	Y10–Y34	Y87.2, Y89.9	N/A
Unintentional exposure to inanimate mechanical forces (firearms)	W32–W34	Y86	N/A
Legal intervention (excluding executions, Y35.5)	Y35.0–Y35.4, Y35.6, Y35.7	Y89.0	N/A
**Abbreviation:** N/A = not applicable.			

Variables analyzed in NVDRS include

- manner of death (i.e., the intent [homicide, legal intervention, suicide, unintentional, or undetermined] of the person on whom a fatal injury was inflicted);
- mechanism of injury (i.e., the method used to inflict a fatal injury) (Box 2);
  BOX 2 **Methods used to inflict injury — National Violent Death Reporting System, 32 states, 2016**
  - Firearm: method that uses a powder charge to fire a projectile from the weapon (excludes BB gun, pellet gun, and compressed air-powered or gas-powered gun)
  - Hanging/strangulation/suffocation (e.g., hanging by the neck, manual strangulation, or plastic bag over the head)
  - Poisoning (e.g., fatal ingestion of a street drug, pharmaceutical, carbon monoxide, gas, rat poison, or insecticide)
  - Sharp instrument (e.g., knife, razor, machete, or pointed instrument)
  - Blunt instrument (e.g., club, bat, rock, or brick)
  - Fall: being pushed or jumping
  - Motor vehicle (e.g., car, bus, motorcycle, or other transport vehicle)
  - Personal weapons (e.g., hands, fists, or feet)
  - Drowning: inhalation of liquid (e.g., in bathtub, lake, or other source of water/liquid)
  - Fire/burns: inhalation of smoke or the direct effects of fire or chemical burns
  - Intentional neglect: starvation, lack of adequate supervision, or withholding of health care
  - Other (single method): any method other than those already listed (e.g., electrocution, exposure to environment/weather, or explosives)
  - Unknown: method not reported or not known
- toxicology findings (for decedents who were tested);
- circumstances preceding injury (i.e., the events that preceded and were identified by investigators as relevant and therefore might have contributed to the infliction of a fatal injury) (Box 3);
  BOX 3 **Circumstances preceding fatal injury, by manner of death — National Violent Death Reporting System, 32 states, 2016**
  **Suicide/Undetermined Intent**
  - Intimate partner problem: decedent was experiencing problems with a current or former intimate partner.
  - Suicide of family member or friend: decedent was distraught over, or reacting to, the suicide of a family member or friend.
  - Other death of family member or friend: decedent was distraught over, or reacting to, the recent nonsuicide death of a family member or friend.
  - Physical health problem: decedent was experiencing physical health problems (e.g., a recent cancer diagnosis or chronic pain).
  - Job problem: decedent was either experiencing a problem at work or having a problem with joblessness.
  - Recent criminal legal problem: decedent was facing criminal legal problems (e.g., recent or impending arrest or upcoming criminal court date).
  - Noncriminal legal problem: decedent was facing civil legal problems (e.g., a child custody or civil lawsuit).
  - Financial problem: decedent was experiencing problems (e.g., bankruptcy, overwhelming debt, or foreclosure of a home or business).
  - Eviction or loss of home: decedent was experiencing a recent or impending eviction or other loss of housing or the threat of eviction or loss of housing.
  - School problem: decedent was experiencing a problem related to school (e.g., poor grades, bullying, social exclusion at school, or performance pressures).
  - Traumatic anniversary: the incident occurred on or near the anniversary of a traumatic event in the decedent’s life.
  - Exposure to disaster: decedent was exposed to a disaster (e.g., earthquake or bombing).
  - Left a suicide note: decedent left a note, e-mail message, video, or other communication indicating intent to die by suicide.
  - Disclosed suicide intent: decedent had recently expressed suicidal feelings to another person with time for that person to intervene.
  - Disclosed intent to whom: type of person (e.g., family member or current or former intimate partner) to whom the decedent recently disclosed suicidal thoughts/plans.
  - History of suicidal thoughts or plans: decedent had previously expressed suicidal thoughts or plans.
  - History of suicide attempt: decedent had previously attempted suicide before the fatal incident.
  **Homicide/Legal Intervention**
  - Jealousy (lovers’ triangle): jealousy or distress over an intimate partner’s relationship or suspected relationship with another person.
  - Stalking: pattern of unwanted harassing or threatening tactics by either the decedent or suspect.
  - Prostitution: prostitution or related activity that includes prostitutes, pimps, clients, or others involved in such activity.
  - Drug involvement: drug dealing, drug trade, or illegal drug use.
  - Brawl: mutual physical fight involving three or more persons.
  - Mercy killing: decedent wished to die because of terminal or hopeless disease or condition, and documentation indicates that the decedent wanted to be killed.
  - Victim was a bystander: decedent was not the intended target in the incident (e.g., pedestrian walking past a gang fight).
  - Victim was a police officer on duty: decedent was a law enforcement officer killed in the line of duty.
  - Victim was an intervener assisting a crime victim: decedent was attempting to assist a crime victim at the time of the incident (e.g., a child attempts to intervene and is killed while trying to assist a parent who is being assaulted).
  - Victim used a weapon: decedent used a weapon to attack or defend during the course of the incident.
  - Intimate partner violence–related: incident is related to conflict between current or former intimate partners; includes the death of an intimate partner or nonintimate partner (e.g., child, parent, friend, or law enforcement officer) killed in an incident that originated in a conflict between intimate partners.
  - Hate crime: decedent was selected intentionally because of his or her actual or perceived gender, religion, sexual orientation, race/ethnicity, or disability.
  - Mentally ill suspect: suspect’s attack on decedent was believed to be the direct result of a mental illness.
  - Drive-by shooting: suspect drove near the decedent and fired a weapon while driving.
  - Walk-by assault: decedent was killed by a targeted attack (e.g., ambush) where the suspect fled on foot.
  - Random violence: decedent was killed in a random act of violence (i.e., an act in which the suspect is not concerned with who is being harmed, just that someone is being harmed).
  - Gang-related: incident resulted from gang activity or gang rivalry; not used if the decedent was a gang member and the death did not appear to result from gang activity.
  - Justifiable self-defense: decedent was killed by a law enforcement officer in the line of duty or by a civilian in legitimate self-defense or in defense of others.
  **All Manners of Death (Except Unintentional Firearm)**
  - Current depressed mood: decedent was perceived by self or others to be feeling depressed at the time of death.
  - Current diagnosed mental health problem: decedent was identified as having a mental health disorder or syndrome listed in the *Diagnostic and Statistical Manual of Mental Disorders, Fourth Edition* (*DSM-IV*), with the exception of alcohol and other substance dependence (which are captured in separate variables).
  - Type of mental health diagnosis: identifies the type of *DSM-IV* diagnosis reported for the decedent.
  - Current mental health treatment: decedent was receiving mental health treatment as evidenced by a current prescription for a psychotropic medication, visit or visits to a mental health professional, or participation in a therapy group within the previous 2 months.
  - History of ever being treated for mental health problem: decedent was identified as having ever received mental health treatment.
  - Alcohol problem: decedent was perceived by self or others to have a problem with, or to be addicted to, alcohol.
  - Substance abuse problem (excludes alcohol): decedent was perceived by self or others to have a problem with, or be addicted to, a substance other than alcohol.
  - Other addiction: decedent was perceived by self or others to have an addiction other than to alcohol or other substance abuse (e.g., gambling or sex).
  - Family relationship problem: decedent was experiencing problems with a family member (other than an intimate partner).
  - Other relationship problem (nonintimate): decedent was experiencing problems with a friend or associate (other than an intimate partner or family member).
  - History of child abuse/neglect: as a child, decedent had history of physical, sexual, or psychological abuse; physical (including medical or dental), emotional, or educational neglect; exposure to a violent environment; or inadequate supervision by a caretaker.
  - Caretaker abuse/neglect led to death: decedent was experiencing physical, sexual, or psychological abuse; physical (including medical or dental), emotional, or educational neglect; exposure to a violent environment; or inadequate supervision by a caretaker that led to death.
  - Perpetrator of interpersonal violence during previous month: decedent perpetrated interpersonal violence during the previous month.
  - Victim of interpersonal violence during previous month: decedent was the target of interpersonal violence during the previous month.
  - Physical fight (two persons, not a brawl): a physical fight between two persons that resulted in the death of the decedent, who was either involved in the fight, a bystander, or trying to stop the fight.
  - Argument or conflict: a specific argument or disagreement led to the victim’s death.
  - Precipitated by another crime: incident occurred as the result of another serious crime.
  - Nature of crime: identifies the specific type of other crime that occurred during the incident (e.g., robbery or drug trafficking).
  - Crime in progress: another serious crime was in progress at the time of the incident.
  - Terrorist attack: decedent was injured in a terrorist attack, leading to death.
  - Crisis during previous or upcoming 2 weeks: current crisis or acute precipitating event or events that either occurred during the previous 2 weeks or was impending in the following 2 weeks (e.g., a trial for a criminal offense begins the following week). Crises typically are associated with specific circumstance variables (e.g., job problem was a crisis or financial problem was a crisis).
  - Other crisis: a crisis related to a death but not captured by any of the standard circumstances.
  **Unintentional Firearm Death**
  *Context of Injury*
  - Hunting: death occurred any time after leaving home for a hunting trip and before returning home from a hunting trip.
  - Target shooting: shooter was aiming for a target and unintentionally hit the decedent; can be at a shooting range or an informal backyard setting (e.g., teenagers shooting at signposts on a fence).
  - Loading/unloading gun: gun discharged when the shooter was loading or unloading ammunition.
  - Cleaning gun: shooter pulled trigger or gun discharged while cleaning, repairing, assembling, or disassembling gun.
  - Showing gun to others: gun was being shown to another person when it discharged or the trigger was pulled.
  - Playing with gun: shooter was playing with a gun when it discharged.
  - Celebratory firing: shooter fired gun in celebratory manner (e.g., firing into the air at midnight on New Year’s Eve).
  - Other context of injury: shooting occurred during some context other than those already described.
  *Mechanism of Injury*
  - Unintentionally pulled trigger: shooter unintentionally pulled the trigger (e.g., while grabbing the gun or holding it too tightly).
  - Thought gun safety was engaged: shooter thought the safety was on and gun would not discharge.
  - Thought unloaded/magazine disengaged: shooter thought the gun was unloaded because the magazine was disengaged.
  - Thought gun was unloaded: shooter thought the gun was unloaded for other unspecified reason.
  - Bullet ricocheted: bullet ricocheted from its intended target and struck the decedent.
  - Gun fired due to defect or malfunction: gun had a defect or malfunctioned as determined by a trained firearm examiner.
  - Gun fired while holstering: gun was being replaced or removed from holster or clothing.
  - Gun was dropped: gun discharged when it was dropped.
  - Gun fired while operating safety/lock: shooter unintentionally fired the gun while operating the safety/lock.
  - Gun was mistaken for toy: gun was mistaken for a toy and was fired without the user understanding the danger.
  - Other mechanism of injury: shooting occurred as the result of a mechanism not already described.
- whether the decedent was a victim (i.e., a person who died as a result of a violence-related injury) or both a suspect and a victim (i.e., a person believed to have inflicted a fatal injury on a victim who then was fatally injured, such as the perpetrator of a homicide-suicide);
- information about any known suspects (i.e., a person or persons believed to have inflicted a fatal injury on a victim);
- incident (i.e., an occurrence in which one or more persons sustained a fatal injury that was linked to a common event or perpetrated by the same suspect or suspects during a 24-hour period); and
- type of incident (i.e., a combination of the manner of death and the number of victims in an incident).

NVDRS is an incident-based system, and all decedents associated with a given incident are grouped in one record. Decisions about whether two or more deaths are related and belong to the same incident are made on the basis of the timing of the injuries rather than on the timing of the deaths. Deaths resulting from injuries that occur within 24 hours of each other and are clearly linked by source documents (discussed under Manner of Death) would be considered part of the same incident. Examples of an incident include 1) a single isolated violent death, 2) two or more related homicides (including legal intervention deaths) when the fatal injuries were inflicted <24 hours apart, 3) two or more related suicides or deaths of undetermined intent when the fatal injuries were inflicted <24 hours apart, and 4) a homicide followed by a suicide when both fatal injuries were inflicted <24 hours apart (*7*).

Information collected from each data source is entered into the NVDRS web-based data entry system (*2*). This system streamlines data abstraction by allowing abstractors to enter data from multiple sources into the same incident record. Internal validation checks, hover-over features that define selected fields, and other quality control measures are included. Primacy rules and hierarchal algorithms related to the source documents occur at the state level. CDC provides access to the web-based system to each state, district, and territory. State project personnel are provided ongoing coding training to learn and adhere to CDC guidance regarding coding of all variables and technical assistance to help increase data quality. Data are transmitted continuously via the web to a CDC-based server. No personally identifiable information is shared with CDC.

### Manner of Death

A manner (i.e., intent) of death for each decedent is assigned by a trained abstractor who integrates information from all source documents. The abstractor-assigned manner of death must agree with at least one required data source; typically, all source documents are consistent regarding the manner of death. When a discrepancy exists, the abstractor must assign a manner of death on the basis of a preponderance of evidence in the source documents; however, such occurrences are rare (*6*). For example, if two sources report a death as a suicide and a third reports it as a death of undetermined intent, the death is coded as a suicide.

NVDRS data are categorized into five abstractor-assigned manners of death: 1) suicide, 2) homicide, 3) unintentional firearm, 4) undetermined intent, and 5) legal intervention.

- **Suicide.** A suicide is a death of a person aged ≥10 years resulting from the use of force against oneself when a preponderance of evidence indicates that the use of force was intentional. This category also includes the following scenarios: 1) deaths of persons who intended only to injure rather than kill themselves; 2) persons who initially intended to kill themselves, changed their minds, but died as a result of the acts; 3) deaths associated with risk-taking behavior without clear intent to inflict fatal self-injury but associated with high risk for death (e.g., playing Russian roulette); 4) suicides that occurred while under the influence of substances or drugs that were taken voluntarily; 5) suicides that occurred while under the influence of a mental illness (e.g., experiencing an acute episode of mental illness); and 6) suicides involving another person providing only passive assistance to the decedent (e.g., supplying the means or information needed to complete the act). This category does not include deaths caused by chronic or acute substance abuse without the intent to die, deaths attributed to autoerotic behavior (e.g., self-strangulation during sexual activity), or assisted suicides (legal or nonlegal). Corresponding *ICD-10* codes included in NVDRS are X60–X84, Y87.0, and U03 (Box 1).
- **Homicide.** A homicide is a death resulting from the use of physical force or power, threatened or actual, against another person, group, or community when a preponderance of evidence indicates that the use of force was intentional. Two special scenarios that CDC’s National Center for Health Statistics (NCHS) regards as homicides are included in the NVDRS case definition: 1) arson with no specified intent to injure someone and 2) a stabbing with intent unspecified. This category also includes the following scenarios: 1) a death when the suspect intended to only injure rather than kill the victim; 2) a death resulting from a heart attack induced when the suspect used force or power against the victim; 3) a death that occurs when a person kills an attacker in self-defense; 4) a death resulting from a weapon that discharges unintentionally while being used to control or frighten a victim; 5) a death attributed to child abuse without intent being specified; 6) a death attributed to an intentional act of neglect by one person against another; 7) a death of an infant that resulted from a direct injury due to violence sustained before birth, and 8) a death identified as justifiable homicide when the person committing homicide was not a law enforcement officer. This category excludes vehicular homicides without intent to injure, unintentional poisoning deaths due to illegal or prescription drug overdose even when a person who provided drugs was charged with homicide, unintentional firearm deaths (a separate category in NVDRS), combat deaths or acts of war, deaths of unborn fetuses, and deaths of infants that resulted indirectly from violence sustained by the mother before birth (e.g., death from prematurity following premature labor brought on by violence). Corresponding *ICD-10* codes included in NVDRS are X85–X99, Y00–Y09, Y87.1, and U01–U02 (Box 1).
- **Unintentional firearm.** An unintentional firearm death is a death resulting from a penetrating injury or gunshot wound from a weapon that uses a powder charge to fire a projectile and for which a preponderance of evidence indicates that the shooting was not directed intentionally at the decedent. Examples include the following: 1) a person who dies as a result of a celebratory firing that was not intended to frighten, control, or harm anyone; 2) a person who unintentionally shoots himself when using a firearm to frighten, control, or harm another person; 3) a soldier who is shot during a field exercise but not in a combat situation; 4) a person who received a self-inflicted wound while playing with a firearm; 5) a person who mistakenly believes a gun is unloaded and shoots another person; 6) a child aged <6 years who shoots himself or another person; and 7) a child who dies after birth from an unintentional firearm injury that was sustained in utero. This category excludes injuries caused by unintentionally striking a person with the firearm (e.g., hitting a person on the head with the firearm rather than firing a projectile) and unintentional injuries from nonpowder guns (e.g., BB, pellet, or other compressed air-powered or gas-powered guns). Corresponding *ICD-10* codes included in NVDRS are W32–W34 and Y86 (Box 1).
- **Undetermined intent.** A death of undetermined intent is a death resulting from the use of force or power against oneself or another person for which the evidence indicating one manner of death is no more compelling than evidence indicating another. This category includes coroner/medical examiner rulings (e.g., accident or suicide, undetermined, jumped or fell, or self-inflicted injury or undetermined intent) where records from data providers indicate that investigators did not find enough evidence to determine whether the injury was intentional. Corresponding *ICD-10* codes included in NVDRS are Y10–Y34, Y87.2, and Y89.9 (Box 1).
- **Legal intervention.** A death from legal intervention is a death in which a person is killed or died as a result of injuries inflicted by a law enforcement officer or other peace officer (i.e., a person with specified legal authority to use deadly force), including military police, while acting in the line of duty. The term legal intervention is a classification from *ICD-10* (Y-35.0) and does not denote the lawfulness or legality of the circumstances surrounding the death. Legal intervention deaths also include a small subset of cases in which force was applied without clear lethal intent (e.g., during restraint or when applying force with a typically nondeadly weapon, such as a Taser) or in which the death occurred while the person was fleeing capture. This category excludes legal executions. Corresponding *ICD-10* codes included in NVDRS are Y35.0–Y35.4, Y35.6, Y35.7, and Y89.0 (Box 1).

### Variables Analyzed

NVDRS collects approximately 600 unique variables for each death. The number of variables recorded for each incident depends on the content and completeness of the source documents. Variables include manner of death; demographic information; *ICD-10* cause of death codes and text descriptors; location, date, and time of injury and death; toxicology results; bodily injuries; precipitating circumstances; victim-suspect relationship and other suspect characteristics; and method of injury (Boxes 1, 2, and 3).

### Circumstances Preceding Death

Circumstances preceding death are defined as the precipitating events that contributed to the infliction of a fatal injury (Box 3). The circumstances are reported on the basis of the content of coroner/medical examiner and law enforcement investigative reports. Certain circumstances are coded to a specific manner of death (e.g., suicide or death of undetermined intent); other circumstances are coded across all manners of death. The data abstractor selects from a list of potential circumstances and is required to code all circumstances that are known to relate to each incident. If circumstances are not known (e.g., for a body found in the woods with no other details reported), the data abstractor leaves the circumstances known variable blank; these deaths are excluded from the denominator for circumstance values. If either the coroner/medical examiner report or law enforcement report indicates the presence of a circumstance, then the abstractor endorses the circumstance (e.g., if the law enforcement report indicated that a decedent had disclosed an intent to die by suicide, then disclosed suicidal intent is endorsed).

### Coding Training and Quality Control

Ongoing coding support for data abstractors is provided through a help desk, monthly conference calls, annual in-person meetings that include coding training for data abstractors, monthly coding work group calls in which all state data abstractors are invited to participate, and regular conference calls with individual states. States also can conduct additional abstractor training workshops and activities at their own discretion, including the use of NVDRS data abstractor e-learning training modules. An NVDRS coding manual (*7*) with CDC-issued standard guidance on coding criteria and examples for each data element is provided. Software features to enhance coding reliability include automated validation rules and a hover-over feature containing variable-specific information.

States are requested to perform annual blind reabstractions of a subset of cases using multiple abstractors to identify inconsistencies. Before releasing data each year, CDC also runs a quality control analysis in which multiple variables are reviewed for their appropriateness, with special focus on abstractor-assigned variables (e.g., method and manner of death). If CDC finds inconsistencies, the state is notified and asked for a response or correction. To ensure no duplicate records are in the final data set, NVDRS first uses SAS (version 9.4; SAS Institute) to search for any instances of duplicates of a unique identification variable associated with each decedent record. As a second and final check for duplicates, the SAS data set is created with an index that only executes successfully if no duplicates of this identification variable are found.

### Time Frame

States are required to begin entering all deaths into the web-based system within 6 months from the date the violent death occurred. States then have an additional 18 months from the end of the calendar year in which the violent death occurred to complete each incident record. Although states typically meet these requirements, additional details sometimes arrive after a deadline has passed. New incidents also might be identified after the deadline (e.g., a death certificate is revised, new evidence is obtained that changes a manner of death, or an *ICD-10* miscoding is corrected to meet NVDRS inclusion criteria). These additional data are incorporated into NVDRS. Analysis files are updated in real time in the web-based system. On the basis of a recent examination of the past 10 data years, CDC estimates that case counts are not likely to increase >1% after the 18-month data collection period.

### Fatal Injuries in 2016

This report provides data on fatal injuries meeting the NVDRS case definition for violent deaths in 2016 that were received by CDC as of September 24, 2018. The 32 participating states used vital statistics death certificate files or coroner/medical examiner reports to identify violent deaths meeting NVDRS case definitions. Each state reported violent deaths of residents that occurred within the state and those of nonresidents for whom a fatal injury occurred within the state (i.e., occurrent deaths). When a violent death was identified, NVDRS data abstractors linked source documents, linked deaths within each incident, coded data elements, and wrote brief narratives of the incident. State data quality was also evaluated by CDC. States meeting minimum or higher data quality standards, such as having descriptive and circumstance data for a sufficient percentage of cases, are included in the NVDRS analytic data set used for this and all other analyses using NVDRS data. All eligible states met this threshold for 2016. State-level data were then consolidated and analyzed.

Numbers, percentages, and crude rates are presented in aggregate for all deaths by abstractor-assigned manner of death. Rates for cells with frequency <20 are not reported because of the instability of those rates (*7*). Illinois, Pennsylvania, and Washington collected data on ≥80% of violent deaths in their state, in accordance with requirements under which the state was funded. Denominators for the rates for these three states represent only the populations of the counties from which data were collected. Rates could not be calculated for certain variables (e.g., precipitating circumstances) because denominators were unknown. Bridged-race 2016 population estimates were used as denominators in the crude rate calculations (*8*). For compatible numerators for rate calculations to be derived, records listing multiple races were recoded to a single race, when possible, using race-bridging methods described by NCHS (available at https://www.cdc.gov/nchs/nvss/bridged_race.htm) (*9*).

## Results

### All Deaths Captured by NVDRS

The 32 NVDRS states included in this report collected data on 40,374 incidents and 41,466 deaths that occurred in 2016. Suicides (n = 25,850; 62.3%) accounted for the highest rate of violent deaths (15.7 per 100,000 population aged ≥10 years), followed by homicides (n = 10,336; 24.9%) (5.5 per 100,000 population). Deaths of undetermined intent (n = 4,470; 10.8%), legal intervention deaths (n = 515; 1.2%), and unintentional firearm deaths (n = 295; <1.0%) occurred at lower rates (2.4, 0.3, and 0.2 per 100,000 population, respectively).

### Suicides

#### Sex, Race/Ethnicity, and Age Group

In 2016, the 32 NVDRS states included in this report collected data on 25,809 incidents involving suicides, which included 25,850 suicide deaths among persons aged ≥10 years. Overall, the suicide rate was 15.7 per 100,000 population. The overall rate for males was 3.5 times the rate for females (24.8 and 7.0 per 100,000 population, respectively) (Table 1); however, the rate for males ranged from 1.9 to 15.9 times the rate for females across age groups and 2.6 to 4.2 times the rate for females across racial/ethnic groups. Adults aged 45–54 years and 55–64 years (19.7 and 18.3 per 100,000 population, respectively) had the highest rate of suicides across age groups. Youths aged 10–19 years accounted for <6% of all suicides and had the lowest rate among all age groups; however, suicide was the second leading cause of death for this age group in 2016 (*1*). Non-Hispanic whites accounted for the majority (82.8%) of suicides. Non-Hispanic American Indians/Alaska Natives had the highest rate of suicides (28.6 per 100,000 population).

**TABLE 1:** Number, percentage,* and rate^†^ of suicides among persons aged ≥10 years,^§^ by decedent’s sex, age group, race/ethnicity, method used, and location in which injury occurred — National Violent Death Reporting System, 32 states,^¶^ 2016

Characteristic	Male		Female		Total	
	No. (%)	Rate	No. (%)	Rate	No. (%)	Rate
Age group (yrs)						
10–14	168 (<1.0)	2.8	90 (1.5)	1.5	**258 (<1.0)**	**2.2**
15–19	927 (4.6)	14.8	291 (4.9)	4.8	**1,218 (4.7)**	**9.9**
20–24	1,781 (8.9)	26.8	398 (6.8)	6.3	**2,179 (8.4)**	**16.8**
25–29	1,760 (8.8)	26.6	393 (6.7)	6.1	**2,153 (8.3)**	**16.5**
30–34	1,595 (8.0)	25.4	470 (8.0)	7.5	**2,065 (8.0)**	**16.5**
35–44	3,051 (15.3)	26.4	1,006 (17.1)	8.6	**4,057 (15.7)**	**17.4**
45–54	3,624 (18.2)	29.3	1,326 (22.5)	10.4	**4,950 (19.1)**	**19.7**
55–64	3,378 (16.9)	28.5	1,125 (19.1)	8.8	**4,503 (17.4)**	**18.3**
65–74	1,913 (9.6)	24.5	529 (9.0)	5.9	**2,442 (9.4)**	**14.6**
75–84	1,190 (6.0)	33.6	190 (3.2)	4.1	**1,380 (5.3)**	**16.8**
≥85	573 (2.9)	44.5	71 (1.2)	2.8	**644 (2.5)**	**17.0**
Unknown	1 (<1.0)	—**	0 (0.0)	—	**1 (<1.0)**	**—**
**Race/Ethnicity**						
White, non-Hispanic	16,542 (82.9)	29.7	4,867 (82.6)	8.4	**21,409 (82.8)**	**18.8**
Black, non-Hispanic	1,278 (6.4)	12.5	340 (5.8)	3.0	**1,618 (6.3)**	**7.5**
American Indian/Alaska Native, non-Hispanic	331 (1.7)	42.8	123 (2.1)	15.1	**454 (1.8)**	**28.6**
Asian/Pacific Islander	522 (2.6)	12.1	220 (3.7)	4.6	**742 (2.9)**	**8.2**
Hispanic^††^	1,232 (6.2)	13.2	311 (5.3)	3.4	**1,543 (6.0)**	**8.4**
Other	56 (<1.0)	—	28 (<1.0)	—	**84 (<1.0)**	**—**
Unknown	0 (0.0)	—	0 (0.0)	—	**0 (0.0)**	**—**
**Method**						
Firearm	11,046 (55.3)	13.7	1,714 (29.1)	2.0	**12,760 (49.4)**	**7.8**
Hanging/strangulation/suffocation	5,534 (27.7)	6.9	1,660 (28.2)	2.0	**7,194 (27.8)**	**4.4**
Poisoning	1,795 (9.0)	2.2	1,940 (32.9)	2.3	**3,735 (14.4)**	**2.3**
Fall	497 (2.5)	0.6	185 (3.1)	0.2	**682 (2.6)**	**0.4**
Sharp instrument	437 (2.2)	0.5	92 (1.6)	0.1	**529 (2.0)**	**0.3**
Motor vehicle (e.g., car, bus, motorcycle, other transport vehicle)	329 (1.6)	0.4	139 (2.4)	0.2	**468 (1.8)**	**0.3**
Drowning	153 (<1.0)	0.2	106 (1.8)	0.1	**259 (1.0)**	**0.2**
Fire/burns	75 (<1.0)	0.1	26 (<1.0)	<0.1	**101 (<1.0)**	**0.1**
Blunt instrument	6 (<1.0)	—	1 (<1.0)	—	**7 (<1.0)**	**—**
Personal weapons (e.g., hands, feet, fists)	1 (<1.0)	—	1 (<1.0)	—	**2 (<1.0)**	**—**
Intentional neglect	0 (0.0)	—	1 (<1.0)	—	**1 (<1.0)**	**—**
Other (single method)	41 (<1.0)	—	11 (<1.0)	—	**52 (<1.0)**	**—**
Unknown	47 (<1.0)	—	13 (<1.0)	—	**60 (<1.0)**	**—**
**Location**						
House/apartment	14,423 (72.3)	18.0	4,702 (79.8)	5.6	**19,125 (74.0)**	**11.6**
Natural area	1,126 (5.6)	1.4	192 (3.3)	0.2	**1,318 (5.1)**	**0.8**
Motor vehicle	1,054 (5.3)	1.3	229 (3.9)	0.3	**1,283 (5.0)**	**0.8**
Street/highway	492 (2.5)	0.6	103 (1.7)	0.1	**595 (2.3)**	**0.4**
Hotel/motel	433 (2.2)	0.5	140 (2.4)	0.2	**573 (2.2)**	**0.3**
Parking lot/public garage/public transport	353 (1.8)	0.4	74 (1.3)	0.1	**427 (1.7)**	**0.3**
Other location^§§^	1,878 (9.4)	—	372 (6.3)	—	**2,250 (8.7)**	**—**
Unknown	202 (1.0)	—	77 (1.3)	—	**279 (1.1)**	**—**
**Total**	**19,961 (100)**	**24.8**	**5,889 (100)**	**7.0**	**25,850 (100)**	**15.7**

* Percentages might not total 100% due to rounding.

^†^ Per 100,000 population.

^§^ Suicide is not reported for decedents aged <10 years, as per standard in the suicide prevention literature. Denominators for the suicide rates represent the total population aged ≥10 years.

^¶^ Alaska, Arizona, Colorado, Connecticut, Georgia, Hawaii, Illinois, Indiana, Iowa, Kansas, Kentucky, Maine, Maryland, Massachusetts, Michigan, Minnesota, New Hampshire, New Jersey, New Mexico, New York, North Carolina, Ohio, Oklahoma, Oregon, Pennsylvania, Rhode Island, South Carolina, Utah, Vermont, Virginia, Washington, and Wisconsin. Illinois, Pennsylvania, and Washington collected data on ≥80% of violent deaths in the state, in accordance with requirements under which the state was funded. Denominators for the rates for these three states represent only the populations of the counties from which the data were collected.

** Rates are not reported when number of decedents is <20 or when characteristic response is “other” or “unknown.”

^††^ Includes persons of any race.

^§§^ Other location includes (in descending order): park/playground/sports or athletic area, jail/prison, railroad tracks, bridge, commercial/retail area, farm, hospital or medical facility, supervised residential facility, cemetery/graveyard/other burial ground, preschool/school/college/school bus, industrial or construction area, office building, abandoned house/building/warehouse, synagogue/church/temple, bar/nightclub, and other unspecified location.

Among males, half (50.4%) of suicide decedents were adults aged 35–64 years. Men aged ≥85 years had the highest rate, followed by men aged 75–84 and 45–54 years (44.5, 33.6, and 29.3 per 100,000 population, respectively) (Table 1). Non-Hispanic American Indian/Alaska Native males had the highest rate of suicides (42.8 per 100,000 population), followed by non-Hispanic white males (29.7 per 100,000 population). The rate of suicide for non-Hispanic American Indian/Alaska Native males was approximately 3.5 times the rate for males with the lowest rate, Asians/Pacific Islanders (12.1 per 100,000 population).

Among females, women aged 35–64 years also accounted for the majority (58.7%) of suicides. Women aged 45–54 years had the highest rate of suicide (10.4 per 100,000 population). Rates were highest for non-Hispanic American Indian/Alaska Native (15.1 per 100,000 population) and non-Hispanic white (8.4 per 100,000 population) females and lowest for non-Hispanic black (3.0 per 100,000 population) and Hispanic (3.4 per 100,000 population) females.

#### Method and Location of Injury

Firearms were used in approximately half (49.4%) of suicides, followed by hanging/strangulation/suffocation (27.8%) and poisoning (14.4%) (7.8, 4.4, and 2.3 per 100,000 population, respectively); the remaining methods used accounted for 8.4% of suicides (Table 1). Among males, the most common method used was a firearm (55.3%), followed by hanging/strangulation/suffocation (27.7%). Among females, poisoning (32.9%), a firearm (29.1%), and hanging/strangulation/suffocation (28.2%) were used in approximately equal proportions. The most common place of suicide was a house/apartment (74.0%) for both males and females (72.3% and 79.8%, respectively), followed by a natural area (5.1%), a motor vehicle (5.0%), a street/highway (2.3%), and a hotel/motel (2.2%).

#### Toxicology Results of Decedent

Tests for alcohol were conducted for 51.5% of suicide decedents (Table 2). Tests for amphetamines, antidepressants, benzodiazepines, cocaine, marijuana, and opioids were conducted for 36.5%, 27.7%, 39.0%, 37.7%, 32.0%, and 40.7% of decedents, respectively. Among those with positive results for alcohol (40.2%), 64.4% of those who tested positive had a blood alcohol concentration (BAC) ≥0.08 g/dL. Results for opioids (including illicit and prescription drugs) were positive in 27.5% of decedents tested for these substances. Results for amphetamines, cocaine, and marijuana were positive in 11.3%, 6.7%, and 23.2% of decedents tested, respectively. Of those tested for antidepressants, 40.1% had positive results at the time of their death, and 30.9% of those tested for benzodiazepines had positive results. Carbon monoxide was tested for in a substantially smaller proportion of decedents (7.7%) but was identified in approximately one third of those decedents (30.7%).

**TABLE 2:** Number* and percentage of suicide decedents who were tested for alcohol and drugs whose results were positive,^†^ by toxicology variables — National Violent Death Reporting System, 32 states,^§^ 2016

Toxicology variable	Tested	Positive
	No. (%)	No. (%)
BAC^¶^	13,310 (51.5)	5,350 (40.2)
Alcohol <0.08 g/dL		1,582 (29.6)
Alcohol ≥0.08 g/dL		3,448 (64.4)
Alcohol positive, level unknown		320 (6.0)
Amphetamines	9,447 (36.5)	1,063 (11.3)
Anticonvulsants	5,572 (21.6)	945 (17.0)
Antidepressants	7,159 (27.7)	2,871 (40.1)
Antipsychotics	5,760 (22.3)	588 (10.2)
Barbiturates	7,915 (30.6)	207 (2.6)
Benzodiazepines	10,070 (39.0)	3,107 (30.9)
Carbon monoxide	2,002 (7.7)	615 (30.7)
Cocaine	9,738 (37.7)	654 (6.7)
Marijuana	8,269 (32.0)	1,916 (23.2)
Muscle relaxants	5,836 (22.6)	435 (7.5)
Opioids	10,532 (40.7)	2,896 (27.5)
Other drugs/substances**	6,005 (23.2)	4,722 (78.6)

**Abbreviation:** BAC = blood alcohol concentration.

* N = 25,850.

^†^ Percentage is of decedents tested for toxicology variable.

^§^ Alaska, Arizona, Colorado, Connecticut, Georgia, Hawaii, Illinois, Indiana, Iowa, Kansas, Kentucky, Maine, Maryland, Massachusetts, Michigan, Minnesota, New Hampshire, New Jersey, New Mexico, New York, North Carolina, Ohio, Oklahoma, Oregon, Pennsylvania, Rhode Island, South Carolina, Utah, Vermont, Virginia, Washington, and Wisconsin. Illinois, Pennsylvania, and Washington collected data on ≥80% of violent deaths in the state, in accordance with requirements under which the state was funded.

^¶^ BAC ≥0.08 g/dL is over the legal limit for impaired driving.

** Other drugs/substances indicated if any results were positive; levels for these drugs/substances are not measured.

#### Precipitating Circumstances

Precipitating circumstances were known for 23,630 (91.4%) of suicide decedents (Table 3). Overall, mental health problems were the most common circumstance, with 49.0% of decedents described as having a current diagnosed mental health problem, 36.9% as experiencing a depressed mood at the time of their death, and 27.0% as currently receiving mental health treatment. Among the 11,577 decedents with a current diagnosed mental health problem, depression/dysthymia (74.0%), anxiety disorder (18.4%), and bipolar disorder (14.2%) were the most common diagnoses.

**TABLE 3:** Number* and percentage^†^ of suicides among persons aged ≥10 years,^§^ by precipitating circumstances and decedent’s sex — National Violent Death Reporting System, 32 states,^¶^ 2016

Precipitating circumstance	Male	Female	Total
	No. (%)	No. (%)	No. (%)
**Mental health/Substance abuse**			
Current diagnosed mental health problem**	8,044 (44.3)	3,533 (64.3)	**11,577 (49.0)**
Depression/dysthymia	5,900 (73.3)	2,665 (75.4)	**8,565 (74.0)**
Anxiety disorder	1,327 (16.5)	802 (22.7)	**2,129 (18.4)**
Bipolar disorder	980 (12.2)	665 (18.8)	**1,645 (14.2)**
Schizophrenia	455 (5.7)	169 (4.8)	**624 (5.4)**
PTSD	461 (5.7)	129 (3.7)	**590 (5.1)**
ADD/ADHD	211 (2.6)	55 (1.6)	**266 (2.3)**
OCD	48 (<1.0)	10 (<1.0)	**58 (<1.0)**
Eating disorder	1 (<1.0)	23 (<1.0)	**24 (<1.0)**
Other	534 (6.6)	184 (5.2)	**718 (6.2)**
Unknown	704 (8.8)	314 (8.9)	**1,018 (8.8)**
Current depressed mood	6,745 (37.2)	1,983 (36.1)	**8,728 (36.9)**
History of ever being treated for a mental health problem	5,886 (32.5)	2,803 (51.0)	**8,689 (36.8)**
Current mental health treatment	4,180 (23.0)	2,209 (40.2)	**6,389 (27.0)**
Alcohol problem	3,360 (18.5)	838 (15.3)	**4,198 (17.8)**
Substance abuse problem (excludes alcohol)	2,930 (16.2)	1,030 (18.8)	**3,960 (16.8)**
Other addiction (e.g., gambling, sex)	170 (<1.0)	24 (<1.0)	**194 (<1.0)**
**Interpersonal**			
Intimate partner problem	5,253 (29.0)	1,391 (25.3)	**6,644 (28.1)**
Family relationship problem	1,576 (8.7)	667 (12.1)	**2,243 (9.5)**
Other death of family member or friend	1,074 (5.9)	391 (7.1)	**1,465 (6.2)**
Suicide of family member or friend	407 (2.2)	153 (2.8)	**560 (2.4)**
Perpetrator of interpersonal violence during past month	511 (2.8)	47 (<1.0)	**558 (2.4)**
Other relationship problem (nonintimate)	390 (2.2)	133 (2.4)	**523 (2.2)**
Victim of interpersonal violence during past month	44 (<1.0)	53 (<1.0)	**97 (<1.0)**
**Life stressor**			
Crisis during previous or upcoming 2 weeks	5,853 (32.3)	1,527 (27.8)	**7,380 (31.2)**
Physical health problem	4,028 (22.2)	1,236 (22.5)	**5,264 (22.3)**
Argument or conflict	2,926 (16.1)	909 (16.6)	**3,835 (16.2)**
Job problem	2,034 (11.2)	376 (6.8)	**2,410 (10.2)**
Financial problem	1,670 (9.2)	382 (7.0)	**2,052 (8.7)**
Recent criminal legal problem	1,771 (9.8)	248 (4.5)	**2,019 (8.5)**
Eviction or loss of home	718 (4.0)	208 (3.8)	**926 (3.9)**
Noncriminal legal problem	635 (3.5)	163 (3.0)	**798 (3.4)**
School problem	288 (1.6)	93 (1.7)	**381 (1.6)**
History of child abuse/neglect	142 (<1.0)	107 (1.9)	**249 (1.1)**
Physical fight (two persons, not a brawl)	203 (1.1)	30 (<1.0)	**233 (<1.0)**
Traumatic anniversary	113 (<1.0)	45 (<1.0)	**158 (<1.0)**
Caretaker abuse/neglect led to death	10 (<1.0)	12 (<1.0)	**22 (<1.0)**
Exposure to disaster	22 (<1.0)	0 (0.0)	**22 (<1.0)**
**Crime and criminal activity**			
Precipitated by another crime	728 (4.0)	60 (1.1)	**788 (3.3)**
Crime in progress^††^	237 (32.6)	16 (26.7)	**253 (32.1)**
Terrorist attack	0 (0.0)	0 (0.0)	**0 (0.0)**
Suicide event			
Left a suicide note	5,776 (31.8)	2,164 (39.4)	**7,940 (33.6)**
History of suicidal thoughts or plans	5,733 (31.6)	1,972 (35.9)	**7,705 (32.6)**
History of suicide attempt	2,990 (16.5)	1,809 (32.9)	**4,799 (20.3)**
**Suicide disclosure**			
Disclosed suicide intent	4,320 (23.8)	1,234 (22.5)	**5,554 (23.5)**
Disclosed intent to whom^§§^			
Previous or current intimate partner	1,659 (38.4)	440 (35.7)	**2,099 (37.8)**
Other family member	1,279 (29.6)	358 (29.0)	**1,637 (29.5)**
Friend/colleague	562 (13.0)	183 (14.8)	**745 (13.4)**
Health care worker	177 (4.1)	72 (5.8)	**249 (4.5)**
Neighbor	40 (<1.0)	14 (1.1)	**54 (<1.0)**
Other person	342 (7.9)	69 (5.6)	**411 (7.4)**
Unknown	261 (6.0)	98 (7.9)	**359 (6.5)**
**Total** ^¶¶^	**18,138 (90.9)**	**5,492 (93.3)**	**23,630 (91.4)**

**Abbreviations:** ADD/ADHD = attention deficit disorder/attention deficit hyperactivity disorder; OCD = obsessive-compulsive disorder; PTSD = posttraumatic stress disorder.

* Includes suicides with one or more precipitating circumstances. More than one circumstance could have been present per decedent.

^†^ Denominator includes those suicides with one or more precipitating circumstances. The sum of percentages in columns exceeds 100% because more than one circumstance could have been present per decedent.

^§^ Suicide is not reported for decedents aged <10 years, as per standard in the suicide prevention literature. Denominators for the suicide rates represent the total population aged ≥10 years.

^¶^ Alaska, Arizona, Colorado, Connecticut, Georgia, Hawaii, Illinois, Indiana, Iowa, Kansas, Kentucky, Maine, Maryland, Massachusetts, Michigan, Minnesota, New Hampshire, New Jersey, New Mexico, New York, North Carolina, Ohio, Oklahoma, Oregon, Pennsylvania, Rhode Island, South Carolina, Utah, Vermont, Virginia, Washington, and Wisconsin. Illinois, Pennsylvania, and Washington collected data on ≥80% of violent deaths in the state, in accordance with requirements under which the state was funded. Denominators for the rates for these three states represent only the populations of the counties from which the data were collected.

** Includes decedents with one or more diagnosed current mental health problems; therefore, sums of percentages for the diagnosed conditions exceed 100%. Denominator includes the number of decedents with one or more current diagnosed mental health problems.

^††^ Denominator includes those decedents involved in an incident that was precipitated by another crime.

^§§^ Denominator includes decedents who disclosed intent.

^¶¶^ Circumstances were unknown for 2,220 decedents (1,823 males and 397 females); total no. of suicide decedents = 25,850 (19,961 males and 5,889 females).

Alcohol or other substance abuse problems were reported for 17.8% and 16.8% of suicide decedents, respectively (Table 3). A crisis during the previous or upcoming 2 weeks (31.2%), intimate partner problems (28.1%), physical health problems (22.3%), and an argument or conflict (16.2%) were among the most commonly reported precipitating circumstances. Among other circumstances related to the suicide event, 33.6% of decedents left a suicide note, 32.6% had a history of suicidal thoughts or plans, 20.3% had a history of previous suicide attempts, and 23.5% had disclosed suicidal intent to another person. Of those who disclosed intent, the greatest proportion of disclosures was to a previous or current intimate partner (37.8%), followed by a family member other than an intimate partner (29.5%).

When examining known circumstances by sex, a greater percentage of female decedents was reported to have had a current diagnosed mental health problem (64.3%) than male decedents (44.3%). Similar percentages of male and female suicide decedents were reported to have had a depressed mood at the time of death (37.2% and 36.1%, respectively). A greater percentage of female (40.2%) than male (23.0%) decedents was known to have been receiving mental health treatment at the time of death. Suicide events, including leaving a suicide note, history of suicidal thoughts and plans, and history of suicide attempts, were reported more frequently for females than males (Table 3).

### Homicides

#### Sex, Race/Ethnicity, and Age Group

The 32 NVDRS states included in this report collected data on 9,692 incidents involving homicides, which included 10,336 homicide deaths in 2016. Overall, the homicide rate was 5.5 per 100,000 population.

Homicide rates for males aged 15–24 years ranged from 5.4 to 6.8 times the rate for females across age groups; however, rates were similar for males and females among persons aged <1–14 years and ≥85 years (Table 4). Non-Hispanic black males accounted for 61.2% of homicides among males and approximately half (n = 5,010; 48.5%) of all homicides and had the highest rate of homicide across any racial/ethnic group (41.5 per 100,000 population). This rate was 13.8 times the homicide rate for non-Hispanic white males (3.0 per 100,000 population), 2.7 times the homicide rate for American Indian/Alaska Native males (15.4 per 100,000 population), and approximately 5.0 times the rate for Hispanic males (8.6 per 100,000 population). The homicide rate for infants aged <1 year was 3.3 times the rate for children aged 1–4 years (7.0 and 2.1 per 100,000 population, respectively). Among female homicide decedents, the rates were highest among infants aged <1 year (7.0 per 100,000 population) and non-Hispanic blacks (5.9 per 100,000 population). The homicide rate for non-Hispanic black females (5.9 per 100,000 population) was 3.7 times the rate for non-Hispanic white females (1.6 per 100,000 population), 5.4 times the rate for Asian/Pacific Islander females (1.1 per 100,000 population), and approximately 3.0 times the rate for Hispanic females (1.9 per 100,000 population).

**TABLE 4:** Number, percentage,* and rate^†^ of homicides, by decedent’s sex, age group, race/ethnicity, method used, location in which injury occurred, and victim-suspect relationship — National Violent Death Reporting System, 32 states,^§^ 2016

Characteristic	Male		Female		Total	
	No. (%)	Rate	No. (%)	Rate	No. (%)	Rate
**Age group (yrs)**						
<1	80 (<1.0)	6.9	77 (3.6)	7.0	**157 (1.5)**	**7.0**
1–4	99 (1.2)	2.1	92 (4.3)	2.1	**191 (1.8)**	**2.1**
5–9	52 (<1.0)	0.9	37 (1.7)	0.6	**89 (<1.0)**	**0.8**
10–14	42 (<1.0)	0.7	35 (1.6)	0.6	**77 (<1.0)**	**0.6**
15–19	841 (10.3)	13.4	152 (7.1)	2.5	**993 (9.6)**	**8.1**
20–24	1,614 (19.7)	24.3	229 (10.7)	3.6	**1,844 (17.8)**	**14.2**
25–29	1,378 (16.8)	20.8	241 (11.2)	3.7	**1,619 (15.7)**	**12.4**
30–34	1,056 (12.9)	16.8	208 (9.7)	3.3	**1,264 (12.2)**	**10.1**
35–44	1,359 (16.6)	11.7	354 (16.5)	3.0	**1,713 (16.6)**	**7.3**
45–54	828 (10.1)	6.7	289 (13.4)	2.3	**1,117 (10.8)**	**4.4**
55–64	498 (6.1)	4.2	217 (10.1)	1.7	**715 (6.9)**	**2.9**
65–74	228 (2.8)	2.9	108 (5.0)	1.2	**336 (3.3)**	**2.0**
75–84	88 (1.1)	2.5	65 (3.0)	1.4	**153 (1.5)**	**1.9**
≥85	20 (<1.0)	1.6	42 (2.0)	1.7	**62 (<1.0)**	**1.6**
Unknown	3 (<1.0)	—^¶^	3 (<1.0)	—	**6 (<1.0)**	**—**
**Race/Ethnicity**						
White, non-Hispanic	1,892 (23.1)	3.0	1,040 (48.4)	1.6	**2,932 (28.4)**	**2.3**
Black, non-Hispanic	5,010 (61.2)	41.5	781 (36.3)	5.9	**5,792 (56.0)**	**22.9**
American Indian/Alaska Native, non-Hispanic	141 (1.7)	15.4	46 (2.1)	4.8	**187 (1.8)**	**10.0**
Asian/Pacific Islander	104 (1.3)	2.1	60 (2.8)	1.1	**164 (1.6)**	**1.6**
Hispanic**	998 (12.2)	8.6	214 (10.0)	1.9	**1,212 (11.7)**	**5.3**
Other	40 (<1.0)	—	8 (<1.0)	—	**48 (<1.0)**	**—**
Unknown	1 (<1.0)	—	0 (0.0)	—	**1 (<1.0)**	**—**
**Method**						
Firearm	6,453 (78.8)	7.0	1,170 (54.4)	1.2	**7,623 (73.8)**	**4.1**
Sharp instrument	761 (9.3)	0.8	337 (15.7)	0.4	**1,098 (10.6)**	**0.6**
Personal weapons (e.g., hands, feet, fists)	290 (3.5)	0.3	133 (6.2)	0.1	**423 (4.1)**	**0.2**
Blunt instrument	271 (3.3)	0.3	140 (6.5)	0.1	**412 (4.0)**	**0.2**
Hanging/strangulation/suffocation	106 (1.3)	0.1	166 (7.7)	0.2	**272 (2.6)**	**0.1**
Motor vehicle (e.g., car, bus, motorcycle, other transport vehicle)	63 (<1.0)	0.1	34 (1.6)	<0.1	**97 (<1.0)**	**0.1**
Fire/burns	46 (<1.0)	<0.1	41 (1.9)	<0.1	**87 (<1.0)**	**<0.1**
Intentional neglect	14 (<1.0)	—	26 (1.2)	<0.1	**40 (<1.0)**	**<0.1**
Poisoning	21 (<1.0)	<0.1	19 (<1.0)	—	**40 (<1.0)**	**<0.1**
Fall	20 (<1.0)	<0.1	3 (<1.0)	—	**23 (<1.0)**	**<0.1**
Drowning	8 (<1.0)	—	8 (<1.0)	—	**16 (<1.0)**	**—**
Other (single method)	29 (<1.0)	—	23 (1.1)	—	**52 (<1.0)**	**—**
Unknown	104 (1.3)	—	49 (2.3)	—	**153 (1.5)**	**—**
**Location**						
House/apartment	3,402 (41.6)	3.7	1,490 (69.3)	1.6	**4,892 (47.3)**	**2.6**
Street/highway	2,306 (28.2)	2.5	192 (8.9)	0.2	**2,498 (24.2)**	**1.3**
Motor vehicle	788 (9.6)	0.9	144 (6.7)	0.2	**932 (9.0)**	**0.5**
Parking lot/public garage/public transport	390 (4.8)	0.4	36 (1.7)	<0.1	**426 (4.1)**	**0.2**
Commercial/retail area	279 (3.4)	0.3	28 (1.3)	<0.1	**307 (3.0)**	**0.2**
Natural area	155 (1.9)	0.2	54 (2.5)	0.1	**209 (2.0)**	**0.1**
Other location^††^	596 (7.3)	—	143 (6.7)	—	**739 (7.1)**	**—**
Unknown	270 (3.3)	—	62 (2.9)	—	**333 (3.2)**	**—**
**Relationship of victim to suspect^§§^**						
Acquaintance/friend	1,009 (35.2)	1.1	153 (10.5)	0.2	**1,162 (26.9)**	**0.6**
Spouse/intimate partner (current or former)	215 (7.5)	0.2	727 (50.1)	0.8	**942 (21.8)**	**0.5**
Other person known to victim	519 (18.1)	—	104 (7.2)	—	**623 (14.4)**	**—**
Stranger	460 (16.1)	0.5	99 (6.8)	0.1	**559 (13.0)**	**0.3**
Child	165 (5.8)	0.2	131 (9.0)	0.1	**296 (6.9)**	**0.2**
Other relative	189 (6.6)	—	72 (5.0)	—	**261 (6.0)**	**—**
Parent	117 (4.1)	0.1	119 (8.2)	0.1	**236 (5.5)**	**0.1**
Rival gang member	109 (3.8)	0.1	4 (<1.0)	—	**113 (2.6)**	**0.1**
Other intimate partner involvement^¶¶^	60 (2.1)	—	41 (2.8)	—	**101 (2.3)**	**—**
Victim was law enforcement officer injured in the line of duty	22 (<1.0)	<0.1	1 (<1.0)	—	**23 (<1.0)**	**<0.1**
**Total**	**8,186 (100)**	**8.9**	**2,149 (100)**	**2.3**	**10,336 (100)**	**5.5**

* Percentages might not total 100% due to rounding. Sex was unknown for one decedent.

^†^ Per 100,000 population.

^§^ Alaska, Arizona, Colorado, Connecticut, Georgia, Hawaii, Illinois, Indiana, Iowa, Kansas, Kentucky, Maine, Maryland, Massachusetts, Michigan, Minnesota, New Hampshire, New Jersey, New Mexico, New York, North Carolina, Ohio, Oklahoma, Oregon, Pennsylvania, Rhode Island, South Carolina, Utah, Vermont, Virginia, Washington, and Wisconsin. Illinois, Pennsylvania, and Washington collected data on ≥80% of violent deaths in the state, in accordance with requirements under which the state was funded. Denominators for the rates for these three states represent only the populations of the counties from which the data were collected.

^¶^ Rates not reported when number of decedents is <20 or when characteristic response is “other” or “unknown.”

** Includes persons of any race.

^††^ Other location includes (in descending order): bar/nightclub, hotel/motel, park/playground/sports or athletic area, abandoned house/building/warehouse, jail/prison, hospital or medical facility, supervised residential facility, office building, industrial or construction area, preschool/school/college/school bus, farm, cemetery/graveyard/other burial ground, railroad tracks, synagogue/church/temple, bridge, and other unspecified location.

^§§^ Percentage is based on the number of homicide decedents with a known victim-suspect relationship (n = 4,316; 2,865 male and 1,451 female); victim-suspect relationship was unknown for 6,020 decedents.

^¶¶^ Death was related to intimate partner violence but not between the intimate partners (e.g., a child killed by mother’s boyfriend).

#### Method, Location of Injury, and Victim-Suspect Relationship

Firearms were used in 73.8% of homicides, followed by sharp instruments (10.6%), personal weapons (e.g., hands, feet, or fists; 4.1%), blunt instruments (4.0%), and hanging/strangulation/suffocation (2.6%) (Table 4). No other method was used in >1% of homicides. Firearms were the most common method used in homicides of both males and females (78.8% and 54.4%, respectively); however, the firearm homicide rate for males was 5.8 times the rate for women (7.0 and 1.2 per 100,000 population, respectively). A greater percentage of fatal injuries among females than among males was caused by sharp instruments (15.7% and 9.3%, respectively), personal weapons (6.2% and 3.5%, respectively), blunt instruments (6.5% and 3.3%, respectively), and hanging/strangulation/suffocation (7.7% and 1.3%, respectively). A house/apartment was the most common location of homicide (47.3%), followed by a street/highway (24.2%), a motor vehicle (9.0%), and a parking lot/public garage/public transport (4.1%). A greater proportion of homicides of females than males occurred at a house/apartment (69.3% and 41.6%, respectively), whereas a greater proportion of homicides of males than females occurred on a street/highway (28.2% and 8.9%, respectively).

The relationship of the victim to the suspect was known for 41.8% of homicides. When the relationship was known, the suspect was most often an acquaintance/friend (26.9%), a current or former spouse/intimate partner (21.8%), other person known to the victim (14.4%), or a stranger (13.0%). Half of female decedents with information on the perpetrator were killed by a current or former intimate partner (50.1%); in contrast, only 7.5% of males were killed by a current or former intimate partner. Among male decedents, the suspect was most frequently an acquaintance/friend (35.2%).

#### Precipitating Circumstances

Precipitating circumstances were identified for 79.3% of homicides (Table 5). Approximately one in three homicides with known circumstances was precipitated by an argument or conflict (32.1%). Homicides were commonly precipitated by another crime (31.0%); in 56.6% of those cases, the crime was in progress at the time of the incident. The types of crime most frequently precipitating the homicide were assault/homicide (43.8%), robbery (34.6%), drug trade** (12.5%), burglary (11.9%), motor vehicle theft (3.4%), rape/sexual assault (2.4%), and arson (2.2%) (Supplementary Table S7, https://stacks.cdc.gov/view/cdc/78990). A physical fight between two persons (13.0%) and drug involvement (12.0%) were other common precipitating circumstances. In 15.6% of homicides with known circumstances, intimate partner violence (IPV) was identified as a contributing factor (Table 5).

**TABLE 5:** Number* and percentage^†^ of homicides, by precipitating circumstances and decedent’s sex — National Violent Death Reporting System, 32 states,^§^ 2016

Precipitating circumstance	Male	Female	Total
**No. (%)**	**No. (%)**	**No. (%)**
**Mental health/Substance abuse**			
Substance abuse problem (excludes alcohol)	748 (11.8)	200 (10.7)	**948 (11.6)**
Current diagnosed mental health problem	274 (4.3)	160 (8.6)	**434 (5.3)**
Alcohol problem	244 (3.9)	73 (3.9)	**317 (3.9)**
History of ever being treated for a mental health problem	183 (2.9)	119 (6.4)	**302 (3.7)**
Current mental health treatment	91 (1.4)	77 (4.1)	**168 (2.1)**
Current depressed mood	28 (<1.0)	27 (1.4)	**55 (<1.0)**
Other addiction (e.g., gambling, sex)	13 (<1.0)	2 (<1.0)	**15 (<1.0)**
**Interpersonal**			
Intimate partner violence–related	474 (7.5)	806 (43.2)	**1,280 (15.6)**
Family relationship problem	251 (4.0)	163 (8.7)	**414 (5.1)**
Other relationship problem (nonintimate)	338 (5.3)	68 (3.6)	**406 (5.0)**
Jealousy (lovers’ triangle)	144 (2.3)	74 (4.0)	**218 (2.7)**
Victim of interpersonal violence during past month	66 (1.0)	101 (5.4)	**167 (2.0)**
Perpetrator of interpersonal violence during past month	88 (1.4)	6 (<1.0)	**94 (1.1)**
**Life stressor**			
Argument or conflict	2,096 (33.1)	536 (28.7)	**2,632 (32.1)**
Physical fight (two persons, not a brawl)	925 (14.6)	138 (7.4)	**1,063 (13.0)**
Crisis during previous or upcoming 2 weeks	493 (7.8)	215 (11.5)	**708 (8.6)**
History of child abuse/neglect	62 (<1.0)	49 (2.6)	**111 (1.4)**
**Crime and criminal activity**			
Precipitated by another crime	2,088 (33.0)	450 (24.1)	**2,538 (31.0)**
Crime in progress^¶^	1,212 (58.0)	224 (49.8)	**1,436 (56.6)**
Drug involvement	876 (13.8)	108 (5.8)	**984 (12.0)**
Gang related	545 (8.6)	60 (3.2)	**605 (7.4)**
Terrorist attack	0 (0.0)	0 (0.0)	**0 (0.0)**
**Homicide event**			
Drive-by shooting	630 (10.0)	76 (4.1)	**706 (8.6)**
Walk-by assault	491 (7.8)	37 (2.0)	**528 (6.4)**
Victim used a weapon	438 (6.9)	27 (1.4)	**465 (5.7)**
Caretaker abuse/neglect led to death	186 (2.9)	192 (10.3)	**378 (4.6)**
Mentally ill suspect	125 (2.0)	131 (7.0)	**256 (3.1)**
Random violence	192 (3.0)	48 (2.6)	**240 (2.9)**
Justifiable self-defense	189 (3.0)	6 (<1.0)	**195 (2.4)**
Brawl	166 (2.6)	12 (<1.0)	**178 (2.2)**
Victim was a bystander	102 (1.6)	73 (3.9)	**175 (2.1)**
Victim was an intervener assisting a crime victim	74 (1.2)	14 (<1.0)	**88 (1.1)**
Stalking	18 (<1.0)	28 (1.5)	**46 (<1.0)**
Victim was a police officer on duty	36 (<1.0)	1 (<1.0)	**37 (<1.0)**
Prostitution	14 (<1.0)	22 (1.2)	**36 (<1.0)**
Mercy killing	2 (<1.0)	12 (<1.0)	**14 (<1.0)**
Hate crime	8 (<1.0)	0 (0.0)	**8 (<1.0)**
Total**	**6,326 (77.3)**	**1,866 (86.8)**	**8,192 (79.3)**

* Includes homicides with one or more precipitating circumstances. Total numbers do not equal the sums of the columns because more than one circumstance could have been present per decedent. Circumstances were unknown for 2,144 decedents (1,860 males and 283 females; sex was unknown for one decedent).

^†^ Denominator includes those homicides with one or more precipitating circumstances. The sum of percentages in columns exceeds 100% because more than one circumstance could have been present per decedent.

^§^ Alaska, Arizona, Colorado, Connecticut, Georgia, Hawaii, Illinois, Indiana, Iowa, Kansas, Kentucky, Maine, Maryland, Massachusetts, Michigan, Minnesota, New Hampshire, New Jersey, New Mexico, New York, North Carolina, Ohio, Oklahoma, Oregon, Pennsylvania, Rhode Island, South Carolina, Utah, Vermont, Virginia, Washington, and Wisconsin. Illinois, Pennsylvania, and Washington collected data on ≥80% of violent deaths in the state, in accordance with requirements under which the state was funded.

^¶^ Denominator includes those decedents involved in an incident that was precipitated by another crime.

** Total no. of homicide decedents = 10,336 (8,186 males and 2,149 females).

Among all reported homicide circumstances, IPV accounted for the largest percentage difference by sex. IPV was a known circumstance for approximately 43.2% of homicides among females but only 7.5% of homicides among males (Table 5). An argument or conflict was a factor in 33.1% of homicides among males and 28.7% of homicides among females. Physical fights precipitated 14.6% of homicides among males but only 7.4% among females. Similarly, drug involvement more commonly precipitated homicide among males, contributing to 13.8% of homicides among males and 5.8% among females. Approximately one in 10 homicides of females resulted from caretaker abuse or neglect (10.3%) compared with only 2.9% of homicides among males. Gang-related homicides were more common among males (8.6%) than females (3.2%). Male decedents used a weapon during the incident in 6.9% and female decedents in 1.4% of homicides with known circumstances.

### Legal Intervention Deaths

#### Sex, Race/Ethnicity, and Age Group

The 32 NVDRS states included in this report collected data on 510 legal intervention death incidents, which included 515 deaths in 2016. Almost all legal intervention deaths occurring in 2016 were among males (95.5%) (Table 6). The highest rate was among males aged 30–34 years (1.3 per 100,000 population), followed by those aged 25–29 years (1.2 per 100,000 population), 35–44 years (1.1 per 100,000 population), and 20–24 years (0.9 per 100,000 population). Non-Hispanic white males accounted for half of legal intervention deaths; however, non-Hispanic black males had the highest rate (1.1 per 100,000 population), 2.8 times the rate for non-Hispanic white males (0.4 per 100,000). Of the 492 male decedents, 15.4% were Hispanic; their legal intervention death rate was 0.7 per 100,000 population.

**TABLE 6:** Number, percentage,* and rate^†^ of legal intervention^§^ deaths, by decedent’s sex, age group, race/ethnicity, method used, and location in which injury occurred — National Violent Death Reporting System, 32 states,^¶^ 2016

Characteristic	Male		Female		Total	
	No. (%)	Rate	No. (%)	Rate	No. (%)	Rate
**Age group (yrs)**						
<1	0 (0.0)	—**	0 (0.0)	—	**0 (0.0)**	**—**
1–4	0 (0.0)	—	0 (0.0)	—	**0 (0.0)**	**—**
5–9	0 (0.0)	—	0 (0.0)	—	**0 (0.0)**	**—**
10–14	1 (<1.0)	—	0 (0.0)	—	**1 (<1.0)**	**—**
15–19	20 (4.1)	0.3	0 (0.0)	—	**20 (3.9)**	**0.2**
20–24	57 (11.6)	0.9	4 (17.4)	—	**61 (11.8)**	**0.5**
25–29	79 (16.1)	1.2	4 (17.4)	—	**83 (16.1)**	**0.6**
30–34	84 (17.1)	1.3	4 (17.4)	—	**88 (17.1)**	**0.7**
35–44	127 (25.8)	1.1	6 (26.1)	—	**133 (25.8)**	**0.6**
45–54	64 (13.0)	0.5	2 (8.7)	—	**66 (12.8)**	**0.3**
55–64	48 (9.8)	0.4	2 (8.7)	—	**50 (9.7)**	**0.2**
65–74	10 (2.0)	—	1 (4.3)	—	**11 (2.1)**	**—**
75–84	2 (<1.0)	—	0 (0.0)	—	**2 (<1.0)**	**—**
≥85	0 (0.0)	—	0 (0.0)	—	**0 (0.0)**	**—**
Unknown	0 (0.0)	—	0 (0.0)	—	**0 (0.0)**	**—**
**Race/Ethnicity**						
White, non-Hispanic	246 (50.0)	0.4	11 (47.8)	—	**257 (49.9)**	**0.2**
Black, non-Hispanic	136 (27.6)	1.1	6 (26.1)	—	**142 (27.6)**	**0.6**
American Indian/Alaska Native, non-Hispanic	19 (3.9)	—	4 (17.4)	—	**23 (4.5)**	**1.2**
Asian/Pacific Islander	14 (2.8)	—	0 (0.0)	—	**14 (2.7)**	**—**
Hispanic^††^	76 (15.4)	0.7	2 (8.7)	—	**78 (15.1)**	**0.3**
Other	1 (<1.0)	—	0 (0.0)	—	**1 (<1.0)**	**—**
Unknown	0 (0.0)	—	0 (0.0)	—	**0 (0.0)**	**—**
**Method**						
Firearm	472 (95.9)	0.5	23 (100)	<0.1	**495 (96.1)**	**0.3**
Motor vehicle (e.g., car, bus, motorcycle, other transport vehicle)	7 (1.4)	—	0 (0.0)	—	**7 (1.4)**	**—**
Drowning	2 (<1.0)	—	0 (0.0)	—	**2 (<1.0)**	**—**
Hanging/strangulation/suffocation	1 (<1.0)	—	0 (0.0)	—	**1 (<1.0)**	**—**
Poisoning	1 (<1.0)	—	0 (0.0)	—	**1 (<1.0)**	**—**
Blunt instrument	0 (0.0)	—	0 (0.0)	—	**0 (0.0)**	**—**
Fall	0 (0.0)	—	0 (0.0)	—	**0 (0.0)**	**—**
Fire/burns	0 (0.0)	—	0 (0.0)	—	**0 (0.0)**	**—**
Intentional neglect	0 (0.0)	—	0 (0.0)	—	**0 (0.0)**	**—**
Personal weapons (e.g., hands, feet, fists)	0 (0.0)	—	0 (0.0)	—	**0 (0.0)**	**—**
Sharp instrument	0 (0.0)	—	0 (0.0)	—	**0 (0.0)**	**—**
Other (single method)	3 (<1.0)	—	0 (0.0)	—	**3 (<1.0)**	**—**
Unknown	6 (1.2)	—	0 (0.0)	—	**6 (1.2)**	**—**
**Location**						
House/apartment	187 (38.0)	0.2	11 (47.8)	—	**198 (38.4)**	**0.1**
Street/highway	143 (29.1)	0.2	3 (13.0)	—	**146 (28.3)**	**0.1**
Motor vehicle	47 (9.6)	0.1	2 (8.7)	—	**49 (9.5)**	**<0.1**
Parking lot/public garage/public transport	28 (5.7)	<0.1	2 (8.7)	—	**30 (5.8)**	**<0.1**
Commercial/retail area	23 (4.7)	<0.1	3 (13.0)	—	**26 (5.0)**	**<0.1**
Other location^§§^	51 (10.4)	—	2 (8.7)	—	**53 (10.3)**	**—**
Unknown	13 (2.6)	—	0 (0.0)	—	**13 (2.5)**	**—**
**Total**	**492 (100)**	**0.5**	**23 (100)**	**<0.1**	**515 (100)**	**0.3**

* Percentages might not total 100% due to rounding.

^†^ Per 100,000 population.

^§^ The term legal intervention does not denote the lawfulness or legality of the circumstances surrounding the death.

^¶^ Alaska, Arizona, Colorado, Connecticut, Georgia, Hawaii, Illinois, Indiana, Iowa, Kansas, Kentucky, Maine, Maryland, Massachusetts, Michigan, Minnesota, New Hampshire, New Jersey, New Mexico, New York, North Carolina, Ohio, Oklahoma, Oregon, Pennsylvania, Rhode Island, South Carolina, Utah, Vermont, Virginia, Washington, and Wisconsin. Illinois, Pennsylvania, and Washington collected data on ≥80% of violent deaths in the state, in accordance with requirements under which the state was funded. Denominators for the rates for these three states represent only the populations of the counties from which the data were collected.

** Rates not reported when number of decedents is <20 or when characteristic response is “other” or “unknown.”

^††^ Includes persons of any race.

^§§^ Other location includes (in descending order): natural area, hotel/motel, park/playground/sports or athletic area, bar/nightclub, hospital or medical facility, preschool/school/college/school bus, abandoned house/building/warehouse, bridge, farm, industrial or construction area, jail/prison, office building, and other unspecified location.

#### Method and Location of Injury

Firearms were used in the majority (96.1%) of legal intervention deaths (Table 6). Legal intervention deaths occurred most frequently in a house/apartment (38.4%), followed by a street/highway (28.3%) or a motor vehicle (9.5%).

#### Precipitating Circumstances

Precipitating circumstances were identified for 98.1% of legal intervention deaths (Table 7). Approximately 90% of legal intervention deaths were precipitated by another serious crime. Among those precipitated by another serious crime (n = 453), assault/homicide (57.0%), robbery (11.0%), motor vehicle theft (7.5%), burglary (4.2%), and drug trade (1.8%) were the types of crimes most frequently precipitating the death (Supplementary Table S9, https://stacks.cdc.gov/view/cdc/78990). In two thirds (65.8%) of legal intervention deaths that were precipitated by another crime, a serious crime was reportedly in progress at the time of the incident; in the remaining cases, a serious crime might have occurred before the incident (Table 7). The decedent reportedly used a weapon in 72.9% of cases. In 25.7% of legal intervention deaths with known circumstances, a substance abuse problem (other than alcohol) was reported as a contributing factor, and 21.0% of decedents reportedly had a current diagnosed mental health problem. An argument or conflict (14.1%), being a perpetrator of interpersonal violence during the past month (9.7%), family relationship problems (6.9%), and drug involvement (5.7%) were other notable precipitating circumstances. Among legal intervention deaths with known circumstances, IPV was identified as a contributing factor in 8.9% of deaths and a crisis during the previous or upcoming 2 weeks was cited in 15.2%.

**TABLE 7:** Number* and percentage^†^ of legal intervention^§^ deaths, by precipitating circumstances and decedent’s sex — National Violent Death Reporting System, 32 states,^¶^ 2016

Precipitating circumstance	Male	Female	Total
	No. (%)	No. (%)	No. (%)
**Mental health/Substance abuse**			
Substance abuse problem (excludes alcohol)	126 (26.1)	4 (18.2)	**130 (25.7)**
Current diagnosed mental health problem	99 (20.5)	7 (31.8)	**106 (21.0)**
History of ever being treated for a mental health problem	73 (15.1)	5 (22.7)	**78 (15.4)**
Alcohol problem	50 (10.4)	2 (9.1)	**52 (10.3)**
Current mental health treatment	36 (7.5)	2 (9.1)	**38 (7.5)**
Current depressed mood	29 (6.0)	2 (9.1)	**31 (6.1)**
Other addiction (e.g., gambling, sex)	3 (<1.0)	0 (0.0)	**3 (<1.0)**
**Interpersonal**			
Perpetrator of interpersonal violence during past month	46 (9.5)	3 (13.6)	**49 (9.7)**
Intimate partner violence-related	45 (9.3)	0 (0.0)	**45 (8.9)**
Family relationship problem	33 (6.8)	2 (9.1)	**35 (6.9)**
Other relationship problem (nonintimate)	15 (3.1)	1 (4.5)	**16 (3.2)**
Jealousy (lovers’ triangle)	3 (<1.0)	0 (0.0)	**3 (<1.0)**
Victim of interpersonal violence during past month	1 (<1.0)	0 (0.0)	**1 (<1.0)**
**Life stressor**			
Crisis during previous or upcoming 2 weeks	73 (15.1)	4 (18.2)	**77 (15.2)**
Argument or conflict	67 (13.9)	4 (18.2)	**71 (14.1)**
Physical fight (two persons, not a brawl)	38 (7.9)	1 (4.5)	**39 (7.7)**
History of child abuse/neglect	2 (<1.0)	0 (0.0)	**2 (<1.0)**
**Crime and criminal activity**			
Precipitated by another crime	435 (90.1)	18 (81.8)	**453 (89.7)**
Crime in progress**	287 (66.0)	11 (61.1)	**298 (65.8)**
Drug involvement	28 (5.8)	1 (4.5)	**29 (5.7)**
Gang-related	4 (<1.0)	0 (0.0)	**4 (<1.0)**
Terrorist attack	0 (0.0)	0 (0.0)	**0 (0.0)**
**Legal intervention event**			
Victim used a weapon	352 (72.9)	16 (72.7)	**368 (72.9)**
Brawl	3 (<1.0)	0 (0.0)	**3 (<1.0)**
Prostitution	2 (<1.0)	0 (0.0)	**2 (<1.0)**
Victim was a bystander	1 (<1.0)	0 (0.0)	**1 (<1.0)**
Victim was a police officer on duty	1 (<1.0)	0 (0.0)	**1 (<1.0)**
Random violence	1 (<1.0)	0 (0.0)	**1 (<1.0)**
Stalking	1 (<1.0)	0 (0.0)	**1 (<1.0)**
Victim was an intervener assisting a crime victim	0 (0.0)	0 (0.0)	**0 (0.0)**
Mentally ill suspect	0 (0.0)	0 (0.0)	**0 (0.0)**
**Total^††^**	**483 (98.2)**	**22 (95.7)**	**505 (98.1)**

* Includes deaths with one or more precipitating circumstances. Total numbers do not equal the sums of the columns because more than one circumstance could have been present per decedent.

^†^ Denominator includes those deaths with one or more precipitating circumstances. The sum of percentages in columns exceed 100% because more than one circumstance could have been present per decedent.

^§^ The term legal intervention does not denote the lawfulness or legality of the circumstances surrounding the death.

^¶^ Alaska, Arizona, Colorado, Connecticut, Georgia, Hawaii, Illinois, Indiana, Iowa, Kansas, Kentucky, Maine, Maryland, Massachusetts, Michigan, Minnesota, New Hampshire, New Jersey, New Mexico, New York, North Carolina, Ohio, Oklahoma, Oregon, Pennsylvania, Rhode Island, South Carolina, Utah, Vermont, Virginia, Washington, and Wisconsin. Illinois, Pennsylvania, and Washington collected data on ≥80% of violent deaths in the state, in accordance with requirements under which the state was funded.

** Denominator includes those decedents involved in an incident that was precipitated by another crime.

^††^ Circumstances were unknown for 10 decedents (nine males and one female); total no. of legal intervention deaths = 515 (492 males and 23 females).

### Unintentional Firearm Deaths

#### Sex, Race/Ethnicity, and Age Group

The 32 NVDRS states included in this report collected data on 293 incidents involving 295 unintentional firearm injury deaths in 2016. Approximately half (n = 151; 51.2%) of these deaths were self-inflicted, and 104 deaths (35.3%) were known to be inflicted by another person; for the remaining 40 deaths (13.6%), the person who inflicted the injury was not known (data not reported). Males accounted for 86.4% of decedents (Table 8). Persons aged ≤24 years accounted for more than half (51.9%) of all unintentional firearm injury deaths. Approximately 17% of decedents were aged <15 years. The majority of decedents were non-Hispanic whites (59.3%), followed by non-Hispanic blacks (29.2%).

**TABLE 8:** Number and percentage* of unintentional firearm deaths, by decedent’s sex, race/ethnicity, age group, location of injury, and type of firearm — National Violent Death Reporting System, 32 states,^†^ 2016

Characteristic	No. (%)
**Sex**	
Male	255 (86.4)
Female	40 (13.6)
**Race/Ethnicity**	
White, non-Hispanic	175 (59.3)
Black, non-Hispanic	86 (29.2)
American Indian/Alaska Native, non-Hispanic	3 (1.0)
Asian/Pacific Islander	2 (<1.0)
Hispanic^§^	27 (9.2)
Other	2 (<1.0)
**Age group (yrs)**	
<1	0 (0.0)
1–4	23 (7.8)
5–9	5 (1.7)
10–14	23 (7.8)
15–19	58 (19.7)
20–24	44 (14.9)
25–29	28 (9.5)
30–34	21 (7.1)
35–44	10 (3.4)
45–54	26 (8.8)
55–64	32 (10.8)
65–74	10 (3.4)
75–84	9 (3.1)
≥85	6 (2.0)
**Location**	
House/apartment	218 (73.9)
Natural area	26 (8.8)
Motor vehicle	13 (4.4)
Street/highway	13 (4.4)
Other location^¶^	17 (5.8)
Unknown	8 (2.7)
**Firearm type**	
Handgun	186 (63.1)
Rifle	32 (10.8)
Shotgun	25 (8.5)
Other firearm	1 (<1.0)
Unknown	51 (17.3)
**Total**	**295 (100)**

* Percentages might not total 100% due to rounding.

^†^ Alaska, Arizona, Colorado, Connecticut, Georgia, Hawaii, Illinois, Indiana, Iowa, Kansas, Kentucky, Maine, Maryland, Massachusetts, Michigan, Minnesota, New Hampshire, New Jersey, New Mexico, New York, North Carolina, Ohio, Oklahoma, Oregon, Pennsylvania, Rhode Island, South Carolina, Utah, Vermont, Virginia, Washington, and Wisconsin. Illinois, Pennsylvania, and Washington collected data on ≥80% of violent deaths in the state, in accordance with requirements under which the state was funded.

^§^ Includes persons of any race.

^¶^ Other location includes (in descending order): commercial/retail area, parking lot/public garage/public transport, abandoned house/building/warehouse, park/playground/sports or athletic area, farm, hotel/motel, and other unspecified location.

#### Location of Injury and Firearm Type

Of all unintentional firearm deaths, 73.9% occurred in a house/apartment, followed by a natural area (8.8%) or a motor vehicle (4.4%) (Table 8). The majority of unintentional firearm injury deaths involved a handgun (63.1%), a rifle (10.8%), or a shotgun (8.5%). In 17.3% of unintentional firearm deaths, the firearm type was unknown.

#### Context of Injury and Associated Circumstances

The context of the injury or associated circumstances was known for 90.8% of unintentional firearm deaths (Table 9). Overall, the most common context of injury was playing with a gun (34.7%), followed by showing the gun to others (12.3%), cleaning the gun (10.1%), and hunting (7.8%). Unintentionally pulling the trigger (23.5%) was the most common associated circumstance, followed by mistakenly thinking the gun was unloaded (9.0%) or mistakenly thinking the magazine was disengaged (6.0%).

**TABLE 9:** Number and percentage* of unintentional firearm deaths, by contexts and circumstances of injury — National Violent Death Reporting System, 32 states,^†^ 2016

Characteristic	No. (%)
**Context of injury**	
Playing with gun	93 (34.7)
Showing gun to others	33 (12.3)
Cleaning gun	27 (10.1)
Hunting	21 (7.8)
Loading/unloading gun	11 (4.1)
Target shooting	7 (2.6)
Celebratory firing	0 (0.0)
Other context of injury	71 (26.5)
**Circumstance of injury**	
Unintentionally pulled trigger	63 (23.5)
Thought gun was unloaded	24 (9.0)
Thought unloaded/magazine disengaged	16 (6.0)
Gun was dropped	15 (5.6)
Gun fired due to defect or malfunction	10 (3.7)
Gun was mistaken for a toy	8 (3.0)
Gun fired while holstering/unholstering	6 (2.2)
Thought gun safety was engaged	5 (1.9)
Bullet ricocheted	3 (1.1)
Gun fired while handling safety/lock	1 (<1.0)
Other mechanism of injury	63 (23.5)
**Total^§^**	**268 (90.8)**

* Percentages might exceed 100% because one or more circumstances could have been known per death; therefore, number and percentage are reported when the number of deaths is <5 because no particular circumstance identifies a single death.

^†^ Alaska, Arizona, Colorado, Connecticut, Georgia, Hawaii, Illinois, Indiana, Iowa, Kansas, Kentucky, Maine, Maryland, Massachusetts, Michigan, Minnesota, New Hampshire, New Jersey, New Mexico, New York, North Carolina, Ohio, Oklahoma, Oregon, Pennsylvania, Rhode Island, South Carolina, Utah, Vermont, Virginia, Washington, and Wisconsin. Illinois, Pennsylvania, and Washington collected data on ≥80% of violent deaths in the state, in accordance with requirements under which the state was funded.

^§^ Circumstances were unknown for 27 decedents; total no. of unintentional firearm decedents = 295.

### Deaths of Undetermined Intent

#### Sex, Race/Ethnicity, and Age Group

The 32 NVDRS states included in this report collected data on 4,436 incidents involving 4,470 deaths for which a determination of intent could not be made (Supplementary Table S1, https://stacks.cdc.gov/view/cdc/78990). The overall rate of deaths of undetermined intent was 2.4 per 100,000 population. The rate was higher among males than among females (3.2 and 1.6 per 100,000 population, respectively) (Supplementary Table S3, https://stacks.cdc.gov/view/cdc/78990). Approximately 70% of persons for whom the manner of death was undetermined were aged 30–64 years. Rates were highest among adults aged 45–54 years (4.0 per 100,000 population), followed by adults aged 30–34 years (3.8 per 100,000 population), 25–29 years (3.7 per 100,000 population), and 35–44 years (3.7 per 100,000 population). Non-Hispanic whites accounted for the majority (71.9%) of deaths, whereas non-Hispanic American Indians/Alaska Natives had the highest rate (4.0 per 100,000 population). Among males, non-Hispanic blacks had the highest rate (5.0 per 100,000 population), followed by non-Hispanic American Indians/Alaska Natives (4.9 per 100,000 population).

#### Method and Location of Injury

Poisoning was the most common method of injury in deaths of undetermined intent (72.2%) (Supplementary Table S3, https://stacks.cdc.gov/view/cdc/78990). No other method accounted for >4% overall. The majority of deaths of undetermined intent occurred in a house/apartment (69.9%), followed by a natural area (4.6%), hotel/motel (3.6%), and street/highway (3.4%).

#### Toxicology Results of Decedent

Tests for antidepressants, benzodiazepines, and opioids were conducted for 40.1%, 44.6%, and 77.4% of decedents, respectively (Supplementary Table S5, https://stacks.cdc.gov/view/cdc/78990). Results for antidepressants and benzodiazepines were positive in 54.2% and 49.9% of decedents tested for those substances, respectively. Results for opioids (including illicit or prescription drugs) were positive in 79.2% of decedents tested.

#### Precipitating Circumstances

Precipitating circumstances were known in 85.6% of deaths of undetermined intent (Supplementary Table S4, https://stacks.cdc.gov/view/cdc/78990). Of those, a current diagnosed mental health problem was reported for 36.7% of decedents; depression/dysthymia (53.6%), anxiety disorder (23.9%), and bipolar disorder (22.8%) were the most common diagnoses. Current depressed mood was reported for 9.9% of decedents, and 21.1% were receiving mental health treatment at the time of their death. Substance abuse problems (other than alcohol) (66.2%) and alcohol problems (28.1%) were the most commonly reported circumstances. Physical health problems (13.0%) and a crisis during the preceding or upcoming 2 weeks (12.0%) were other life stressors identified in deaths of undetermined intent. Among decedents, 10.1% had a history of suicidal thoughts or plans, 8.3% had a history of suicide attempts, and 4.7% had disclosed intent to die by suicide.

### Suicides Among Youths Aged 10–24 Years

#### Sex, Race/Ethnicity, and Age Group

The 32 NVDRS states included in this report collected data on 3,655 suicides among youths aged 10–24 years that occurred during 2016 (Table 10). Of these decedents, 911 (24.9%) were aged 10–17 years and 2,744 (75.1%) were aged 18–24 years. The majority of youth decedents were male (78.7%) and non-Hispanic white (68.1%).

**TABLE 10:** Number and percentage* of youth suicides by age group, sex, race/ethnicity, method used, and location in which injury occurred — National Violent Death Reporting System, 32 states,^†^ 2016

Characteristic	10–17 yrs	18–24 yrs	Total
	No. (%)	No. (%)	No. (%)
**Sex**			
Male	632 (69.4)	2,244 (81.8)	**2,876 (78.7)**
Female	279 (30.6)	500 (18.2)	**779 (21.3)**
**Race/Ethnicity**			
White, non-Hispanic	646 (70.9)	1,842 (67.1)	**2,488 (68.1)**
Black, non-Hispanic	84 (9.2)	350 (12.8)	**434 (11.9)**
American Indian/Alaska Native, non-Hispanic	30 (3.3)	99 (3.6)	**129 (3.5)**
Asian/Pacific Islander	37 (4.1)	151 (5.5)	**188 (5.1)**
Hispanic^§^	108 (11.9)	289 (10.5)	**397 (10.9)**
Other	6 (<1.0)	13 (<1.0)	**19 (<1.0)**
**Method**			
Firearm	368 (40.4)	1,267 (46.2)	**1,635 (44.7)**
Hanging/strangulation/suffocation	449 (49.3)	1,025 (37.4)	**1,474 (40.3)**
Poisoning	39 (4.3)	191 (7.0)	**230 (6.3)**
Fall	23 (2.5)	103 (3.8)	**126 (3.4)**
Motor vehicle (e.g., car, bus, motorcycle, other transport vehicle)	23 (2.5)	82 (3.0)	**105 (2.9)**
Drowning	4 (<1.0)	31 (1.1)	**35 (<1.0)**
Sharp instrument	1 (<1.0)	23 (<1.0)	**24 (<1.0)**
Fire/burns	1 (<1.0)	13 (<1.0)	**14 (<1.0)**
Blunt instrument	0 (0.0)	2 (<1.0)	**2 (<1.0)**
Other (single method)	2 (<1.0)	4 (<1.0)	**6 (<1.0)**
Unknown	1 (<1.0)	3 (<1.0)	**4 (<1.0)**
**Location**			
House/apartment	737 (80.9)	1,801 (65.6)	**2,538 (69.4)**
Natural area	50 (5.5)	203 (7.4)	**253 (6.9)**
Motor vehicle	15 (1.6)	168 (6.1)	**183 (5.0)**
Street/highway	12 (1.3)	89 (3.2)	**101 (2.8)**
Park/playground/sports or athletic area	16 (1.8)	74 (2.7)	**90 (2.5)**
Parking lot/public garage/public transport	9 (<1.0)	50 (1.8)	**59 (1.6)**
Preschool/school/college/school bus	11 (1.2)	45 (1.6)	**56 (1.5)**
Other location^¶^	48 (5.3)	284 (10.3)	**332 (9.1)**
Unknown	13 (1.4)	30 (1.1)	**43 (1.2)**
**Total**	**911 (100)**	**2,744 (100)**	**3,655 (100)**

* Percentages might not total 100% due to rounding.

^†^ Alaska, Arizona, Colorado, Connecticut, Georgia, Hawaii, Illinois, Indiana, Iowa, Kansas, Kentucky, Maine, Maryland, Massachusetts, Michigan, Minnesota, New Hampshire, New Jersey, New Mexico, New York, North Carolina, Ohio, Oklahoma, Oregon, Pennsylvania, Rhode Island, South Carolina, Utah, Vermont, Virginia, Washington, and Wisconsin. Illinois, Pennsylvania, and Washington collected data on ≥80% of violent deaths in the state, in accordance with requirements under which the state was funded.

^§^ Includes persons of any race.

^¶^ Other location includes (in descending order): railroad tracks, bridge, hotel/motel, jail/prison, commercial/retail area, farm, supervised residential facility, hospital or medical facility, cemetery/graveyard/other burial ground, office building, abandoned house/building/warehouse, industrial or construction area, synagogue/church/temple, bar/nightclub, and other unspecified location.

#### Method and Location of Injury

The most common method used was a firearm (44.7%), followed by hanging/strangulation/suffocation (40.3%) and poisoning (6.3%) (Table 10). Hanging/strangulation/suffocation was the method used in approximately half (49.3%) of suicides among youths aged 10–17 years compared with 37.4% of suicides among youths aged 18–24 years. The majority (69.4%) of youth suicides occurred in a house/apartment, followed by a natural area (6.9%), a motor vehicle (5.0%), a street/highway (2.8%), and a park/playground/sports or athletic area (2.5%). However, a greater percentage of suicides among youths aged 10–17 years occurred at home than among youths aged 18–24 years (80.9% and 65.6%, respectively), and a greater percentage of suicides among those aged 18–24 years occurred in a motor vehicle compared with those aged 10–17 years (6.1% and 1.6%, respectively).

#### Toxicology Results of Decedent

Tests for alcohol were conducted for approximately half of all suicide decedents aged 10–17 years and 18–24 years (50.8% and 53.2%, respectively) (Table 11). Among those tested, youths aged 18–24 years more commonly tested positive for alcohol compared with youths aged 10–17 years (39.0% and 10.8%, respectively); of those, 64.7% of youths aged 18–24 years had BACs ≥0.08 g/dL compared with 36.0% of youths aged 10–17 years. Among youths tested for antidepressants, 28.3% of those aged 10–17 years tested positive compared with 22.8% among those aged 18–24 years. In contrast, youths aged 18–24 years tested positive for benzodiazepines, marijuana, and opioids more commonly than those aged 10–17 years.

**TABLE 11:** Number* and percentage of youth suicide decedents who were tested for alcohol and drugs whose results were positive,^†^ by age group and toxicology variables — National Violent Death Reporting System, 32 states,^§^ 2016

Toxicology variable	10–17 yrs		18–24 yrs	
	Tested	Positive	Tested	Positive
	No. (%)	No. (%)	No. (%)	No. (%)
BAC^¶^	463 (50.8)	50 (10.8)	1,460 (53.2)	570 (39.0)
Alcohol < 0.08 g/dL		28 (56.0)		178 (31.2)
Alcohol ≥0.08 g/dL		18 (36.0)		369 (64.7)
Alcohol positive, level unknown		4 (8.0)		23 (4.0)
Amphetamines	382 (41.9)	38 (9.9)	1,057 (38.5)	143 (13.5)
Anticonvulsants	204 (22.4)	15 (7.4)	550 (20.0)	51 (9.3)
Antidepressants	258 (28.3)	73 (28.3)	668 (24.3)	152 (22.8)
Antipsychotics	217 (23.8)	5 (2.3)	602 (21.9)	24 (4.0)
Barbiturates	313 (34.4)	3 (<1.0)	868 (31.6)	9 (1.0)
Benzodiazepines	358 (39.3)	31 (8.7)	1,047 (38.2)	206 (19.7)
Carbon monoxide	57 (6.3)	3 (5.3)	186 (6.8)	34 (18.3)
Cocaine	362 (39.7)	2 (<1.0)	1,092 (39.8)	85 (7.8)
Marijuana	335 (36.8)	83 (24.8)	1,006 (36.7)	431 (42.8)
Muscle relaxants	235 (25.8)	2 (<1.0)	610 (22.2)	17 (2.8)
Opioids	377 (41.4)	23 (6.1)	1,108 (40.4)	150 (13.5)
Other drugs/substances**	136 (14.9)	88 (64.7)	514 (18.7)	367 (71.4)

**Abbreviation:** BAC = blood alcohol concentration.

* No. of youth suicide decedents aged 10–17 years = 911 and aged 18–24 years = 2,744.

^†^ Percentage is of decedents tested for toxicology variable.

^§^ Alaska, Arizona, Colorado, Connecticut, Georgia, Hawaii, Illinois, Indiana, Iowa, Kansas, Kentucky, Maine, Maryland, Massachusetts, Michigan, Minnesota, New Hampshire, New Jersey, New Mexico, New York, North Carolina, Ohio, Oklahoma, Oregon, Pennsylvania, Rhode Island, South Carolina, Utah, Vermont, Virginia, Washington, and Wisconsin. Illinois, Pennsylvania, and Washington collected data on ≥80% of violent deaths in the state, in accordance with requirements under which the state was funded.

^¶^ BAC ≥0.08 g/dL is over the legal limit for impaired driving.

** Other drugs/substances indicated if any results were positive; levels for these drugs/substances are not measured.

#### Precipitating Circumstances

Precipitating circumstances were known for 90.1% of suicide decedents aged 10–24 years (Table 12). Of those with known circumstance information, similar proportions of youths aged 10–17 years and 18–24 years had a current diagnosed mental health problem (46.3% and 45.7%, respectively) or were described as being depressed at the time of their death (35.6% and 36.3%, respectively). Among youths aged 10–24 years with a diagnosed mental health problem, depression/dysthymia (70.9%), anxiety disorder (17.6%), and bipolar disorder (12.8%) were the most common diagnoses. Attention deficit disorder/attention deficit hyperactivity disorder was more commonly diagnosed among youths aged 10–17 years than among those aged 18–24 years (16.7% and 5.9%, respectively), whereas bipolar disorder and schizophrenia were more commonly diagnosed among youths aged 18–24 years than among those aged 10–17 years (15.5% versus 4.8% and 6.5% versus <1.0%, respectively). A slightly greater percentage of youths aged 10–17 years was known to be receiving mental health treatment at the time of death than those aged 18–24 years (30.3% and 24.3%, respectively). Substance abuse problems and alcohol problems were reported more often among youths aged 18–24 years than among those aged 10–17 years (21.2% versus 9.1% and 12.4% versus 3.2%, respectively).

**TABLE 12:** Number* and percentage^†^ of youth suicides, by precipitating circumstances and decedent’s age group — National Violent Death Reporting System, 32 states,^§^ 2016

Precipitating circumstance	10–17 yrs	18–24 yrs	Total
	No. (%)	No. (%)	No. (%)
**Mental health/Substance abuse**			
Current diagnosed mental health problem^¶^	377 (46.3)	1,133 (45.7)	**1,510 (45.9)**
Depression/dysthymia	279 (74.0)	792 (69.9)	**1,071 (70.9)**
Anxiety disorder	75 (19.9)	191 (16.9)	**266 (17.6)**
Bipolar disorder	18 (4.8)	176 (15.5)	**194 (12.8)**
ADD/ADHD	63 (16.7)	67 (5.9)	**130 (8.6)**
Schizophrenia	2 (<1.0)	74 (6.5)	**76 (5.0)**
PTSD	6 (1.6)	47 (4.1)	**53 (3.5)**
OCD	1 (<1.0)	11 (<1.0)	**12 (<1.0)**
Eating disorder	1 (<1.0)	5 (<1.0)	**6 (<1.0)**
Other	55 (14.6)	68 (6.0)	**123 (8.1)**
Unknown	32 (8.5)	119 (10.5)	**151 (10.0)**
History of ever being treated for a mental health problem	335 (41.2)	881 (35.6)	**1,216 (36.9)**
Current depressed mood	290 (35.6)	900 (36.3)	**1,190 (36.1)**
Current mental health treatment	247 (30.3)	602 (24.3)	**849 (25.8)**
Substance abuse problem (excludes alcohol)	74 (9.1)	525 (21.2)	**599 (18.2)**
Alcohol problem	26 (3.2)	307 (12.4)	**333 (10.1)**
Other addiction (e.g., gambling, sex)	3 (<1.0)	14 (<1.0)	**17 (<1.0)**
**Interpersonal**			
Intimate partner problem	204 (25.1)	816 (32.9)	**1,020 (31.0)**
Family relationship problem	265 (32.6)	294 (11.9)	**559 (17.0)**
Other relationship problem (nonintimate)	62 (7.6)	94 (3.8)	**156 (4.7)**
Other death of family member or friend	44 (5.4)	109 (4.4)	**153 (4.6)**
Suicide of family member or friend	36 (4.4)	77 (3.1)	**113 (3.4)**
Perpetrator of interpersonal violence during past month	3 (<1.0)	38 (1.5)	**41 (1.2)**
Victim of interpersonal violence during past month	5 (<1.0)	12 (<1.0)	**17 (<1.0)**
**Life stressor**			
Crisis during previous or upcoming 2 weeks	317 (38.9)	781 (31.5)	**1,098 (33.4)**
Argument or conflict	202 (24.8)	536 (21.6)	**738 (22.4)**
School problem	212 (26.0)	117 (4.7)	**329 (10.0)**
Recent criminal legal problem	35 (4.3)	230 (9.3)	**265 (8.0)**
Job problem	6 (<1.0)	216 (8.7)	**222 (6.7)**
Physical health problem	32 (3.9)	104 (4.2)	**136 (4.1)**
Financial problem	4 (<1.0)	127 (5.1)	**131 (4.0)**
History of child abuse/neglect	40 (4.9)	48 (1.9)	**88 (2.7)**
Eviction or loss of home	3 (<1.0)	53 (2.1)	**56 (1.7)**
Noncriminal legal problem	6 (<1.0)	41 (1.7)	**47 (1.4)**
Physical fight (two persons, not a brawl)	9 (1.1)	38 (1.5)	**47 (1.4)**
Traumatic anniversary	5 (<1.0)	16 (<1.0)	**21 (<1.0)**
Caretaker abuse/neglect led to death	5 (<1.0)	4 (<1.0)	**9 (<1.0)**
Exposure to disaster	0 (0.0)	1 (<1.0)	**1 (<1.0)**
**Crime and criminal activity**			
Precipitated by another crime	20 (2.5)	87 (3.5)	**107 (3.3)**
Crime in progress**	6 (30.0)	28 (32.2)	**34 (31.8)**
Terrorist attack	0 (0.0)	0 (0.0)	**0 (0.0)**
**Suicide event**			
History of suicidal thoughts or plans	290 (35.6)	886 (35.8)	**1,176 (35.7)**
Left a suicide note	322 (39.6)	816 (32.9)	**1,138 (34.6)**
History of suicide attempt	173 (21.3)	589 (23.8)	**762 (23.1)**
**Suicide disclosure**			
Disclosed suicide intent	201 (24.7)	652 (26.3)	**853 (25.9)**
Disclosed intent to whom^††^			
Previous or current intimate partner	38 (18.9)	232 (35.6)	**270 (31.7)**
Other family member	57 (28.4)	194 (29.8)	**251 (29.4)**
Friend/colleague	65 (32.3)	120 (18.4)	**185 (21.7)**
Health care worker	7 (3.5)	14 (2.1)	**21 (2.5)**
Other person	22 (10.9)	57 (8.7)	**79 (9.3)**
Unknown	12 (6.0)	35 (5.4)	**47 (5.5)**
**Total^§§^**	**814 (89.4)**	**2,478 (90.3)**	**3,292 (90.1)**

**Abbreviations:** ADD/ADHD = attention deficit disorder/attention deficit hyperactivity disorder; OCD = obsessive-compulsive disorder; PTSD = posttraumatic stress disorder.

* Includes suicides with one or more precipitating circumstances. More than one circumstance could have been present per decedent.

^†^ Denominator includes those suicides with one or more precipitating circumstances. The sum of percentages in columns exceeds 100% because more than one circumstance could have been present per decedent.

^§^ Alaska, Arizona, Colorado, Connecticut, Georgia, Hawaii, Illinois, Indiana, Iowa, Kansas, Kentucky, Maine, Maryland, Massachusetts, Michigan, Minnesota, New Hampshire, New Jersey, New Mexico, New York, North Carolina, Ohio, Oklahoma, Oregon, Pennsylvania, Rhode Island, South Carolina, Utah, Vermont, Virginia, Washington, and Wisconsin. Illinois, Pennsylvania, and Washington collected data on ≥80% of violent deaths in the state, in accordance with requirements under which the state was funded.

^¶^ Includes decedents with one or more diagnosed current mental health problems; therefore, sums of percentages for the diagnosed conditions exceed 100%. Denominator includes the number of decedents with one or more current diagnosed mental health problems.

** Denominator includes those decedents involved in an incident that was precipitated by another crime.

^††^ Denominator includes decedents who disclosed intent.

^§§^ Circumstances were unknown for 363 decedents (97 aged 10–17 years and 266 aged 18–24 years); total no. of youth suicide decedents = 3,655 (911 aged 10–17 years and 2,744 aged 18–24 years).

With respect to interpersonal relationships, intimate partner problems were a common precipitating circumstance among youths aged 10–24 years (31.0%); approximately one in three youths aged 18–24 years (32.9%) and one in four youths aged 10–17 years (25.1%) were known to have an intimate partner problem contribute to their death (Table 12). Family relationship problems were reported for 17.0% of suicides among youths aged 10–24 years; however, among interpersonal suicide circumstances, family relationship problems accounted for the largest percentage difference between youths aged 10–17 years and youths aged 18–24 years (32.6% versus 11.9%, respectively). A crisis during the previous or upcoming 2 weeks was the most frequently documented life stressor for youths aged 10–24 years (33.4%), followed by an argument or conflict (22.4%), school problems (10.0%), and a recent criminal legal problem (8.0%). A crisis was more common among youths aged 10–17 years than among those aged 18–24 years (38.9% versus 31.5%, respectively). Among life stressors, school problems accounted for the largest percentage difference between youths aged 10–17 years and youths aged 18–24 years (26.0% versus 4.7%, respectively).

Among other circumstances related to the suicide event, 35.7% of youths aged 10–24 years had a history of suicidal thoughts or plans, 34.6% left a suicide note, and 23.1% had a history of suicide attempts (Table 12). A greater percentage among youths aged 10–17 years left a suicide note than among those aged 18–24 years (39.6% versus 32.9%, respectively). Approximately one fourth of decedents aged 10–24 years (25.9%) disclosed suicidal intent to another person. Among youths aged 10–17 years who disclosed intent, the greatest proportion of disclosure was to a friend/colleague (32.3%), followed by a family member (28.4%) and a previous or current intimate partner (18.9%). Among youths aged 18–24 years who disclosed intent, the greatest proportion of disclosure was to a previous or current intimate partner (35.6%), followed by a family member (29.8%) and a friend/colleague (18.4%).

## Discussion

Violent deaths affect males and females and persons of all ages, races, and ethnicities. NVDRS data help identify disparities in and characteristics of populations particularly affected by fatal violence. Violence also occurs in different forms that are linked to one another in important ways. NVDRS not only provides details on specific manners of violent death, but also has the capacity to identify cross-cutting risk factors for multiple forms of violence. These details increase knowledge about the circumstances associated with violence and can help public health authorities and their partners develop and guide data-informed, effective approaches to violence prevention.

The occurrence of violent death varies greatly across states (*1*). Whereas this report summarizes 2016 data from 32 U.S. states, in 2019 NVDRS expanded data collection to include all 50 states, the District of Columbia, and Puerto Rico, achieving a truly national NVDRS (*10*). With nationwide expansion, NVDRS will provide more comprehensive, accessible, and actionable violent death information and equip all states and communities with data for appropriate public health action at the local level. The 32 states that provided data for this report accounted for 58.9% of violent deaths and represented 58% of the U.S. population in 2016 (*1*,*4*). The nationwide expansion will allow CDC to provide valuable information on the scope of violent deaths in the United States, provide data for development of evidence-based violence prevention efforts at the national level, and allow every U.S. state to have access to this data at state, local, and regional levels.

Violence is preventable, and reducing violent deaths in communities is possible with evidence-based approaches (*11*). CDC developed technical packages to assist communities and states in identifying violence prevention approaches that are based on the best available evidence (*11*). The five technical packages include strategies; approaches; and specific programs, practices, and policies with evidence of effects on risk for child abuse and neglect, IPV, youth violence, sexual violence, and suicide. Each package considers the multifaceted and interactive effects of individual, relationship, family, school, and community factors that can influence violence-related outcomes. The strategies and approaches are intended to work together across the different levels of social ecology in a multisector effort to prevent violence. Sectors instrumental to the implementation of these packages include education, government, social services, justice, housing, faith-based organizations, and the private sector (*11*).

The findings in this report demonstrate that demographic variations in the manner of death from violence-related injuries persist. Suicides comprised the majority (62.3%) of violent deaths collected in NVDRS and occurred at higher rates among non-Hispanic American Indians/Alaska Natives, followed by non-Hispanic whites. Suicide rates were highest among males and adults aged 45–64 years. Infants experienced homicide rates of 7.0 per 100,000 population, a rate that was 3.3 times the rate among children aged 1–4 years, 8.8 times the rate among children aged 5–9 years, and 11.7 times the rate among youths aged 10–14 years.

The high homicide rate for infants reinforces the need for prioritizing child abuse and neglect prevention and intervention strategies. Creating safe, stable, and nurturing relationships and environments is essential for the prevention of child abuse and neglect. CDC’s child abuse and neglect prevention technical package (*12*) identified the following evidence-based strategies and approaches to help prevent child abuse and neglect: 1) strengthening economic supports for families, 2) changing social norms to support parents and positive parenting, 3) providing quality care and education early in life, 4) enhancing parenting skills to promote healthy child development, and 5) intervening to lessen harms and prevent future risk. Child abuse and neglect is preventable, and the specific approaches described in the technical package combined with creating safe, stable, and nurturing relationships and environments (*13*) can help reduce homicides of children and infants as well as child maltreatment and other adverse childhood experiences.

Homicide rates were highest among persons aged 20–29 years, especially males. For both males and females, rates were highest among non-Hispanic blacks and American Indians/Alaska Natives. Among males, the homicide rate among non-Hispanic blacks was 41.5 per 100,000 population, a rate 2.7 times that for non-Hispanic American Indian/Alaska Native and approximately 14 times that for non-Hispanic white males.

Racial and ethnic minorities experience inequitable rates of violent injury and homicide, and these inequities are pervasive and persistent (*14*). The racial/ethnic inequities in homicide rates identified in this report, particularly among youths and young adult males, warrant prioritizing inequities related to race/ethnicity in violence prevention. These inequities result from the disproportionate exposure of racial and ethnic minorities to systemic inequities such as residential segregation, concentrated disadvantage, stress from experiencing racism, limited access to the best educational and employment opportunities, and other conditions that increase risk for experiencing violence (*14*–*16*). Racial and ethnic minority youths often live in communities with concentrated poverty, stressed economies, residential instability and neighborhood disorganization, access to firearms and illegal drugs, and low community cohesion and informal controls (*15*,*16*). All these conditions are associated with violence and violence-related injuries (*15*). Prevention efforts will achieve greater population-level reductions in violence through the reduction of systemic inequities and by targeting salient neighborhood and community-level contributors to violence (*17*). Evaluations of programs such as Baltimore’s *Safe Streets,* Crime Prevention Through Environmental Design (CPTED), business improvement districts, and policies such as the earned income tax credit (*18*) have confirmed the value in employing these types of community-level strategies in reducing violence. Evidence also suggests that these strategies and other universal policies that focus on general community improvements can have substantial impact on decreasing the race/ethnicity gap in violence (*15*).

A firearm was the most common method used in suicides. Lethal means, such as firearms, provide limited opportunity for intervention and have high case-fatality rates (*19*). Creating protective environments by reducing access to lethal means among persons at risk can be an effective strategy to prevent suicide (*19*). Past suicidal behavior and mental illness are important risk factors for suicide as well (*19*). In this report, 32.6% of suicide decedents had a history of suicidal thoughts or plans and 23.5% had disclosed their suicide intent. These precipitants are well documented as important risk factors to target in suicide prevention (*19*,*20*). Mental health problems are also still the most commonly identified circumstance and continue to be a key focus for prevention. Less than one third of suicide decedents were known to be receiving treatment at the time of death, pointing to a gap between those receiving treatment and those who would likely benefit from it.

Although treatment for mental health problems is an essential component of suicide prevention, previous research with NVDRS data has documented that more than half of suicide decedents did not have a known mental health condition (*21*). That study indicated that multiple factors contribute to suicide among those with and without known mental health conditions. Although mental health conditions are important, other potential opportunities exist for prevention. For example, findings in this report illustrate that intimate partner problems and recent crises were also frequent precipitants of suicide.

CDC’s suicide prevention technical package (*19*) contains the following seven strategies for reducing suicide and suicidal behaviors: 1) strengthen economic supports, 2) strengthen access and delivery of suicide care, 3) create protective environments, 4) promote connectedness, 5) teach coping and problem-solving skills, 6) identify and support persons at risk, and 7) lessen harms and prevent future risk. Each strategy includes examples of specific approaches that states and communities can implement to advance the strategy. This report’s findings indicate that several approaches, including social-emotional learning programs, treatment for persons at risk for suicide and treatment to prevent reattempts, and enhancing parenting skills and family relationships, will be important to include when developing suicide prevention programs. Suicide prevention efforts are best achieved if these approaches and strategies are used in combination and reinforce one another to reduce risk for suicide and have cross-cutting impact on other forms of violence (*19*). By using NVDRS data, suicide prevention experts can guide planning and implementation and track outcomes of suicide prevention strategies and approaches within their states and communities. The strategies in the technical package support the goals and objectives of the National Strategy for Suicide Prevention (NSSP) (*22*) and the National Action Alliance for Suicide Prevention’s priority to strengthen community-based prevention (*23*).

NVDRS homicide circumstance data indicate that homicides among males were most often precipitated by an argument or conflict or during the commission of a crime (predominately assault/homicide). In contrast, 43.2% of homicides among females were related to IPV; a current or former spouse/intimate partner was identified as the perpetrator in half of homicides of women with known perpetrators. These findings were consistent with another NVDRS report that highlighted the differential impact of IPV-related homicides among young and racial and ethnic minority women (*24*).

Efforts to reduce IPV among women include screening women of childbearing age for IPV and referring those who have a positive screen to intervention services (*25*) and providing counseling services for pregnant women experiencing IPV (*26*). IPV screening that is conducted in a culturally sensitive way is important to minimize threats to safety. Strategies and approaches across different levels of the social ecology to prevent IPV also include providing support to survivors; empowering bystanders; engaging men and boys as allies (*27*,*28*); disrupting developmental pathways toward IPV; creating protective home, school, and workplace environments (*27*); and teaching youths about safe and healthy relationships before they begin dating (*27*,*29*,*30*). Evidence-based, cross-cutting prevention efforts that incorporate changing social norms, including harmful gender norms that condone violence, and societal conditions that serve to maintain harmful norms and inequality across sex, race/ethnicity, and income groups are also effective approaches outlined in CDC’s IPV prevention technical package (*27*).

Relationship problems are frequent precipitants of suicides and homicides, underscoring that many forms of violence are interconnected and might share the same root causes (*31*). Therefore, the impact of violence prevention and intervention efforts can be broadened by understanding shared risk and protective factors and addressing multiple, connected forms of violence (*31*). This report’s findings also support the need to implement programs that develop social and emotional skills (e.g., problem solving, conflict resolution, and individual coping skills) and cultivate supportive relationships to protect against violent injuries and death. For example, Safe Dates, a school-based program designed to reduce dating violence among adolescents, has indicated promise for reducing long-term physical and sexual dating violence as well as peer violence victimization and weapon carrying (*27*,*29*,*30*). Furthermore, primary prevention strategies designed to teach skills that reduce aggressive behavior toward others and improve social skills, emotional well-being, and self-esteem can be targeted toward preadolescents and early adolescents before violent behaviors and patterns begin (*17*). CDC’s youth violence technical package emphasizes the preventive effects of skill development programs for youths and prevention approaches that address relationships and influence school and community environments (*17*).

Substance use is another frequent precipitant of suicide and interpersonal violent behavior. Toxicology results documented a high prevalence of alcohol, especially with BACs ≥0.08g/dL, among suicide and homicide decedents tested for substance use. Alcohol use is a robust predictor of suicidal behavior (*32*), victimization (*33*), and interpersonal violence perpetration (*17*,*27*). Intoxication can lead to disinhibition, enhance feelings of hopelessness and depression, and impair judgment that can lead to impulsive behaviors (*21*). Alcohol use can also reduce awareness and perception of surrounding risks, thus increasing one’s vulnerability to being victimized (*34*). Most deaths of undetermined intent were poisonings. Opioids (illicit or prescription) were the most common substances detected in deaths of undetermined intent; among those tested, 79.2% had positive opioid results. Whether these deaths were the result of unintentional drug poisoning or suicide is unknown. Opioid overdose has been recognized as an epidemic (*35*). CDC published *Guideline for Prescribing Opioids for Chronic Pain* to help address the epidemic; support safer prescribing practices; and reduce opioid misuse, opioid use disorder, and overdose (*36*). Other important activities include expanding naloxone availability and access to medication-assisted treatment, enhancing public health and public safety partnerships, and maximizing the ability of health systems to link persons to treatment and harm-reduction services (*37*).

NVDRS also collects more complete information than other data sources on legal intervention deaths (*38*) and unintentional firearm deaths (*39*). Findings from 32 states indicate that the largest proportion of deaths due to legal intervention was among non-Hispanic white males; however, the rate among non-Hispanic black males was approximately three times that of their white male counterparts, a finding consistent with previous studies (*40*,*41*). Further analyses are needed to increase knowledge about the magnitude and circumstances of these deaths, which is essential to developing appropriate prevention strategies and monitoring their effectiveness. NVDRS also has been recognized as a reliable source of data on unintentional firearm deaths (*39*) and for its capability to provide details about victims and shooters (*42*). Findings in this report indicate that more than half of unintentional firearm deaths were self-inflicted, but approximately 35.3% were known to be inflicted by another person. Most of these deaths occurred while playing with a gun, accidentally pulling the trigger, or while thinking the gun was unloaded, particularly among children (*43*), highlighting the importance of safe storage practices and education about safe handling of firearms.

NVDRS data permit examination of violent deaths involving specific populations. Findings in this report indicate that most suicide decedents aged 10–24 years were non-Hispanic white males aged 18–24 years. Approximately half of all youths aged 10–17 years died by hanging/strangulation/suffocation, whereas the proportion of suicides that were from firearm-related injuries was greater among youths aged 18–24 years (46.2%). Among youth suicide decedents aged 10–24 years, approximately half had a diagnosed mental health condition. Depression was the most common diagnosis among all youth suicide decedents. The prevalence of attention deficit disorder/attention deficit hyperactivity disorder was greater among youths aged 10–17 years compared with youths aged 18–24 years (16.7% versus 5.9%, respectively). The most common precipitating circumstances associated with suicide among youths aged 10–24 years were interpersonal problems and a crisis during the previous or upcoming 2 weeks. However, the nature of the relationship problems experienced by older and younger youths differed. Family relationship problems were more commonly a precipitant among youths aged 10–17 years and intimate partner problems were more common among youths aged 18–24 years, reflecting the importance of different relationships at different points in youth development.

Several approaches might be helpful in reducing suicide among youths. Programs that promote connectedness through safe, stable, and nurturing relationships (e.g., Sources of Strength) have been reported to increase perceptions of adult support for suicidal youths and acceptability of help-seeking behaviors among high school students (*44*). Social-emotional learning programs (e.g., Youth Aware of Mental Health Program) (*45*) and parenting skill and family relationship programs (e.g., Strengthening Families 10–14) (*46*) teach coping and problem-solving skills to youths and their families. Youth Aware of Mental Health Program teaches adolescents aged 14–16 years about the risk and protective factors associated with suicide and enhances their problem-solving skills for dealing with adverse life events, stress, school, and other problems (*45*). Strengthening Families 10–14 improves parents’ skills for disciplining, managing emotions and conflict, and communicating with their children; promotes youths’ interpersonal and problem-solving skills; and creates family activities to build cohesion and positive parent-child interactions (*46*). Finally, identifying persons at risk for suicide and delivering treatment and support for these individuals through gatekeeper training can have a positive impact on suicide and its associated risk factors (*19*). Gatekeepers are community members (e.g., teachers, coaches, and primary health care providers) trained to identify persons who might be at risk for suicide and to respond effectively by facilitating treatment seeking and support services (*19*). Counties that implemented gatekeeper training through the Garret Lee Smith Memorial Suicide Prevention Program were found to have significantly lower suicide rates among youths aged 10–24 years 1 year after the training implementation than counties that did not implement the program (*47*).

NVDRS data have been used by states to examine the circumstances surrounding youth suicides and develop suicide prevention programs (*48*–*51*). For example, because the unadjusted suicide rate in Utah for youths aged 10–17 years had more than doubled during 2011–2015, in 2017 the Utah Department of Public Health collaborated with CDC to conduct an epidemiologic investigation of suicides among that age group (*48*). To identify precipitating circumstances for these suicides, data were analyzed from the Utah Violent Death Reporting System (UTVDRS) for years 2015–2017 (*48*). Precipitating circumstances were found to include mental health problems, depressed mood, a history of suicidal ideation, a recent crises such as family and intimate partner problems, school problems, and suicide of a friend or family member. Narrative data from UTVDRS identified further details about the precipitating circumstances. For instance, family conflict resulting from or resulting in the restriction of technology use (e.g., mobile phones, tablets, gaming systems, or laptops) was a factor in approximately 13% of suicides in which circumstances were known. Results of the investigation point to target areas for preventing suicide and promoting mental health. Data describing youth suicide in Utah have been used to support suicide prevention efforts such as the creation of a student safety and crisis tip line, a study focused on prevention of firearm suicides, and a position at the Office of the Medical Examiner to study youth suicide deaths (*49*). Furthermore, the nuanced findings regarding technology restriction as a contributing factor in youth suicides stimulate areas for future research (*48*,*49*).

NVDRS data have been integral in ongoing statewide surveillance on suicide among youths. In Rhode Island, data from the Rhode Island Violent Death Reporting System (RIVDRS) were analyzed to identify precipitating circumstances of youth suicide deaths in the state during 2004–2014 (*50*). RIVDRS data indicated that 127 youths aged ≤25 years had died by suicide in Rhode Island during the 10-year period. Analyses provided information on injury location and weapon type, indicating that most youths died at home by hanging/strangulation/suffocation. RIVDRS data also indicated that the majority (74%) of decedents with a mental health problem at the time of death had a diagnosis of depression and dysthymia. The results prompted the development of Rhode Island’s Suicide Prevention Initiative (SPI) to address the link between suicide and depression. SPI is a referral system that links school crisis team members with emergency service clinicians who provide emergency mental health assessments to youths in elementary, middle, and high schools who are experiencing crises and suicidal ideation (*50*).

Data from NVDRS also have been useful for multisectoral collaborations targeted at understanding and preventing youth suicide in states. The Kansas Youth Suicide Prevention Task Force was convened by the Kansas Attorney General’s office in 2018 to examine statewide efforts to prevent youth suicide and recommend changes in practice, policy, or law aimed at preventing youth suicide in Kansas (*51*). Data from the Kansas Violent Death Reporting System (KSVDRS) were presented at the inaugural meeting of the task force. The KSVDRS presentation encompassed an introduction to NVDRS and information on suicides among Kansas youths aged 10–17 years. KSVDRS staff continue to share information with the task force on the basis of their experience collecting data and reporting on violent death and suicide throughout the state.

NVDRS also is relevant to two national prevention initiatives: NSSP and *Healthy People 2020* (*22*,*52*). NSSP is a comprehensive national agenda for suicide prevention (*22*). In particular, NVDRS is relevant to NSSP goals of increasing timeliness and usefulness of surveillance systems related to suicide prevention and evaluating outcomes and effectiveness of suicide prevention interventions. *Healthy People 2020* includes objectives for reducing the number of suicides, homicides, and firearm-related deaths and increasing the number of states that link data on violent deaths from death certificates, law enforcement reports, and coroner/medical examiner reports at state and local levels (*52*). NVDRS data can be used to measure states’ progress toward these goals by allowing for the examination of changing patterns in circumstances and risk profiles, which is not possible with other data sources.

## Limitations

The findings provided in this report are subject to at least eight limitations. First, NVDRS data are available from a limited number of states and therefore are not nationally representative.

Second, the availability, completeness, and timeliness of data are dependent on partnerships among state Violent Death Reporting System programs and state health departments, vital statistics registrars’ offices, coroners/medical examiners, and law enforcement personnel. Data sharing and communication among partners is particularly challenging when states have independent county coroner systems rather than a centralized coroner/medical examiner system, a large number of law enforcement jurisdictions, or both. NVDRS incident data might be limited or incomplete for areas in which these data-sharing relationships are not developed fully.

Third, toxicology data are not collected consistently across all states or for all alcohol and drug categories. Toxicology testing is not conducted for all decedents, so the percentages of those with positive results for specific substances might be affected by selective testing patterns in coroner/medical examiner offices (*52*).

Fourth, abstractors are limited to the data included in the investigative reports they receive. Reports might not fully reflect all information known about an incident, particularly for homicides and legal intervention deaths, when data are less readily available until after a full investigation and adjudication are completed.

Fifth, case definitions present challenges when a single death is classified differently in different documents (e.g., unintentional in a law enforcement report, homicide in a coroner/medical examiner report, and undetermined on the death certificate). NVDRS abstractors reconcile these discrepancies using standard NVDRS case definitions and select a single manner of death on the basis of all source documents; the manner of death assigned must be consistent with the manner of death noted in at least one source document.

Sixth, variations in coding might occur depending on the abstractor’s level of experience. For this reason, CDC provides extensive abstractor guidance and training and data validation checks, and states conduct blinded reabstractions of cases to test consistency and identify training needs.

Seventh, medical and mental health information (e.g., type of condition and whether the decedent was currently receiving treatment) often are not captured directly from medical records but from coroner/medical examiner reports and the decedent’s family members and friends. Therefore, completeness and accuracy of this information is limited by the knowledge of the informant.

Finally, protective factor data (i.e., characteristics or circumstances that reduce the risk for violent death) are not collected by NVDRS because of the nature of death certificates, coroner/medical examiner reports, and law enforcement reports, which typically contain only circumstances associated with risk factors.

## Conclusion

Public health surveillance is the foundation for public health practice (*53*). Monitoring prevalence and incidence of violence-related fatal injuries, defining priorities, and informing programmatic and violence prevention activities are essential parts of public health surveillance (*54*). In 2018, NVDRS received funding for nationwide expansion. As of 2019, all 50 states, Puerto Rico, and the District of Columbia participate in NVDRS, a move toward achieving the ultimate goal of providing nationally representative data by including all states, all U.S. territories, and the District of Columbia. This expansion will not only make violent death information available for every state to develop local prevention efforts, but will allow for the system’s capacity to measure the need for and effects of violence prevention policies, programs, and practices at the national level.

## Footnotes

* The name of the Institute of Medicine was changed to the National Academy of Medicine, effective July 1, 2015.

† To be included in NVDRS, deaths of undetermined intent must have some evidence of the possibility that the intent was purposeful, including use of a weapon or other evidence that force was used to inflict the injury. Most commonly, the coroner/medical examiner is unsure whether the death was a suicide or unintentional.

§ California began collecting data in 2005 but ended data collection in 2009. In 2017, California resumed data collection.

¶ Frequencies and rates of violent deaths included in this report will differ slightly from the frequencies and rates of violent deaths reported by NVDRS WISQARS, which excludes nonresident deaths that occur in participating states (i.e., occurrent deaths). NVDRS tracks both resident and occurrent violent deaths in the overall data set, and the numbers in this report reflect both. States are expected to collect information on violent deaths among their residents, wherever they occur, and fatal violent injuries occurring within their borders irrespective of the decedent’s residence status. If the states of residence and injury occurrence are both participating NVDRS states, the state of injury occurrence is responsible for collecting the information. By making this differentiation of responsibility, duplicate reporting is avoided.

** Drug trade is defined as the buying, selling, or passing of drugs in exchange for goods or money. Drug involvement includes drug use in addition to drug trade.
